# Prediction of intracranial response to PD-1/PD-L1 inhibitors therapy in brain metastases originating from non-small cell lung cancer using habitat imaging and peritumoral radiomics: a multicenter study

**DOI:** 10.3389/fonc.2025.1657290

**Published:** 2025-10-28

**Authors:** Min Ding, Tianrui He, Jing Yu, Jian Zheng, Song Wei, Yuan Yuan, Chunhui Yang, Ning Luo, Xin Qi, Liting Liu, Yiyang Sun, Dailun Hou, Chao Yang, Hongxu Liu, Wenwen Liu, Qi Wang

**Affiliations:** ^1^ Department of Respiratory Medicine, The Second Affiliated Hospital of Dalian Medical University, Dalian, China; ^2^ Postgraduate College, Dalian Medical University, Dalian, China; ^3^ Zhongshan Hospital Institute of Clinical Science, Fudan University Shanghai Medical College, Shanghai, China; ^4^ Department of Thoracic Oncology, Cancer Hospital of Dalian University of Technology, Liaoning Cancer Hospital and Institute, Cancer Hospital of China Medical University, Shenyang, China; ^5^ Department of Medical Oncology, Beijing Chest Hospital, Capital Medical University and Beijing Tuberculosis and Thoracic Tumor Research Institute, Beijing, China; ^6^ Department of Clinical Laboratory, The Second Hospital of Dalian Medical University, Dalian, China; ^7^ Department of Radiology, The Second Affiliated Hospital of Dalian Medical University, Dalian, China; ^8^ Department of Radiology, The First Affiliated Hospital of Dalian Medical University, Dalian, China; ^9^ Beijing Chest Hospital, Capital Medical University, Beijing, China; ^10^ Department of Thoracic Surgery, Cancer Hospital of Dalian University of Technology, Liaoning Cancer Hospital and Institute, Shenyang, China; ^11^ Cancer Translational Medicine Research Center, The Second Hospital, Dalian Medical University, Dalian, China

**Keywords:** radiomics, habitat imaging, PD-1/PD-L1 inhibitors, lung cancer, brain metastasis

## Abstract

**Background:**

Predicting the intracranial efficacy of programmed death-1/programmed death-ligand 1 (PD-1/PD-L1) inhibitors in non-small cell lung cancer (NSCLC) patients with brain metastasis (BM) remains challenging. The objective of this study was to construct a habitat-peritumoral radiomics framework for immunotherapy response prediction, concurrently identifying the optimal peritumoral extent.

**Methods:**

This retrospective multicenter study analyzed 378 NSCLC-BM patients receiving PD-1/PD-L1 inhibitors. Participants were stratified into training (n=146), internal validation (n=63), and two external test cohorts (test 1: n=57; test 2: n=112). Logistic regression was conducted to determine significant clinical predictors. Habitat subregion segmentation was performed using K-means clustering with peritumoral extensions at incremental distances (1, 2, and 3 mm). Predictive models were developed using radiomic features extracted from intratumoral cores, habitat subregions, and peritumoral zones through machine learning approaches. A combined model integrated habitat signatures, peritumoral features, and clinical predictors. Model performance assessment employed the area under the curves (AUCs), calibration curves, and decision curve analyses (DCA).

**Results:**

The habitat-based XGBoost model demonstrated superior predictive performance across all cohorts compared to alternative models, achieving AUCs of 0.900 (training), 0.886 (internal validation), 0.820 (test 1), and 0.804 (test 2). For peritumoral analysis, the peri-1 mm RandomForest model exceeded other regional configurations. Integrating peri-1 mm features and clinical factors yielded a marginal performance enhancement in the combined model, with corresponding AUCs of 0.898, 0.894, 0.837, and 0.814. The combined model demonstrated optimal calibration and significant clinical utility, as evidenced by calibration curves and DCA.

**Conclusion:**

The validated habitat-peritumoral radiomics framework, optimized at a 1-mm peritumoral extent, demonstrates robust predictive accuracy for intracranial immunotherapy response in NSCLC-BM patients and offers significant clinical utility.

## 1 Introduction

Non-small cell lung cancer (NSCLC) maintains its status among the foremost causes of worldwide cancer mortality (1). Brain metastasis (BM) develops frequently as a complication in advanced disease cohorts, affecting up to 40% of patients (2). These intracranial lesions significantly compromise neurological function and survival outcomes, underscoring the urgent need for effective intracranial disease control (3). Programmed death-1/programmed death-ligand 1 (PD-1/PD-L1) inhibitors have emerged as pivotal therapeutic options for NSCLC. However, their intracranial efficacy exhibits marked heterogeneity (4, 5). In a phase II clinical trial, pembrolizumab yielded a 29.7% intracranial objective response rate among 40 untreated brain-metastatic NSCLC patients (6). Spatial heterogeneity across tumor sites and procedural challenges in tissue sampling diminish the reliability of traditional predictive biomarkers such as PD-L1 and tumor mutational burden (TMB) for BM assessment (7). Consequently, developing non-invasive techniques to predict PD-1/PD-L1 inhibitors efficacy among NSCLC-BM patients represents an imperative neurooncological priority (8, 9).

Magnetic resonance imaging (MRI) serves a pivotal role in BM diagnosis and therapeutic monitoring (10, 11). As non-invasive predictive tools, MRI radiomics have shown clinical utility in predicting intracranial immunotherapy responses among brain-metastatic lung cancer patients in earlier investigations (12). However, these studies typically extracted features from either the entire tumor volume or its combination with peritumoral regions. This approach fundamentally assumes feature homogeneity within such volumes of interest, overlooking regional phenotypic variability within BM (13). Consequently, it inevitably fails to capture the spatial heterogeneity between distinct intratumoral subregions, thereby discarding potentially valuable information for predicting immunotherapy efficacy (14).

Habitat imaging, a radiomics methodology that leverages tumor microenvironments, partitions lesions into discrete subregions (habitats) with distinct phenotypic characteristics reflecting histopathologic variations (15, 16). Distinct tumor habitats may exhibit unique growth and invasion patterns, along with potentially differential therapeutic responses (17). Moreover, the peritumoral microenvironment significantly influences tumor development and advancement. Combining intratumoral and peritumoral data enables a multidimensional assessment of intracranial treatment efficacy.

**Figure 1:**
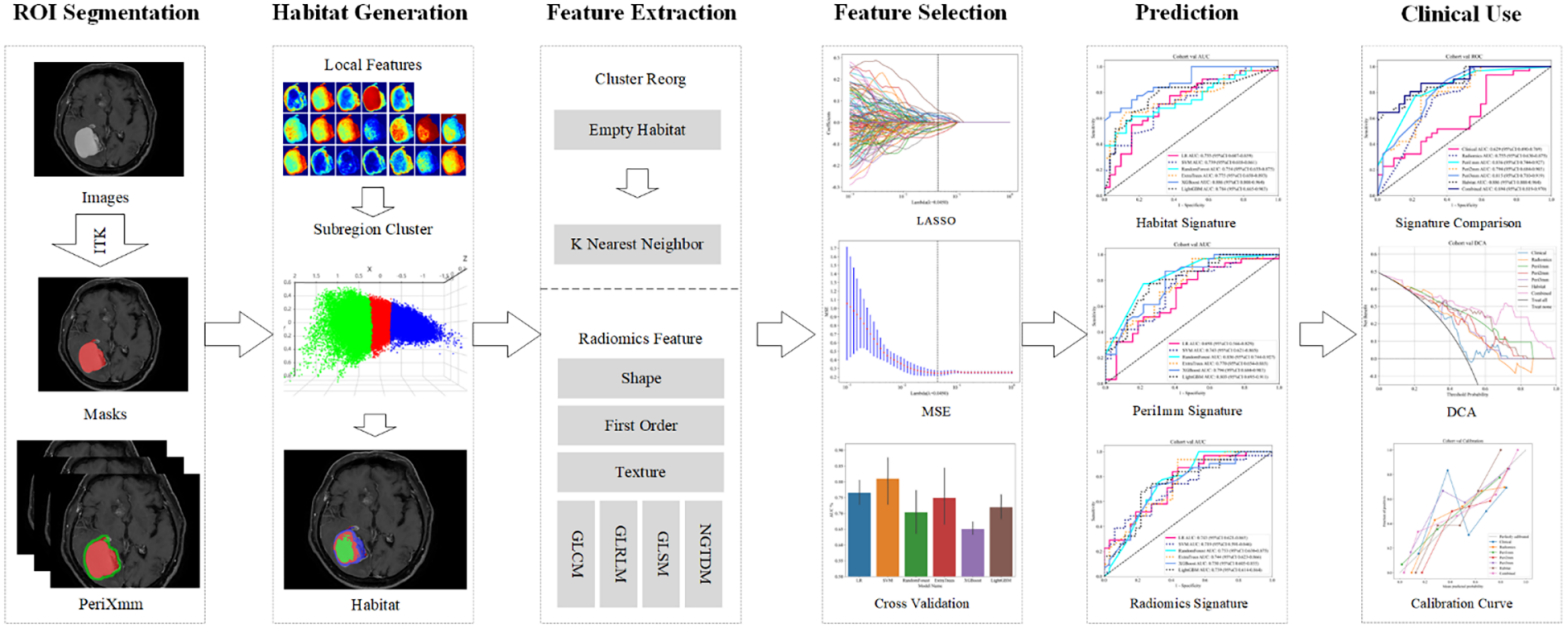
Overall workflow of this study. ROI, region of interest; LASSO, least absolute shrinkage and selection operator; MSE, mean squared error; DCA, decision curve analysis.

Through multicenter analysis, voxel-wise clustering enabled habitat subregion development that mapped intratumoral heterogeneity, concurrently revealing the superior peritumoral boundary for predictive capability improvement. Based on this framework, we constructed a multimodal integrated model that incorporates habitat features, peritumoral characteristics, and clinical factors. This model delivers a non-invasive clinical decision aid to identify NSCLC-BM patients deriving maximal therapeutic benefit from PD-1/PD-L1 inhibitors. The overall research process of our study is as shown in **Figure 1**.

## 2 Methods

Due to the retrospective nature of this study, the ethics committee granted a waiver for informed consent, and the study was conducted in strict accordance with the principles outlined in the Declaration of Helsinki.

### 2.1 Patient selection and data collection

This retrospective cohort comprised 1,156 lung cancer patients with BMs receiving PD-1/PD-L1 inhibitors across four institutions from January 2017 to September 2024: Second Affiliated Hospital of Dalian Medical University (n=199), Liaoning Cancer Hospital (n=454), First Affiliated Hospital of Dalian Medical University (n=110), and Beijing Chest Hospital (n=393).

**Figure 2:**
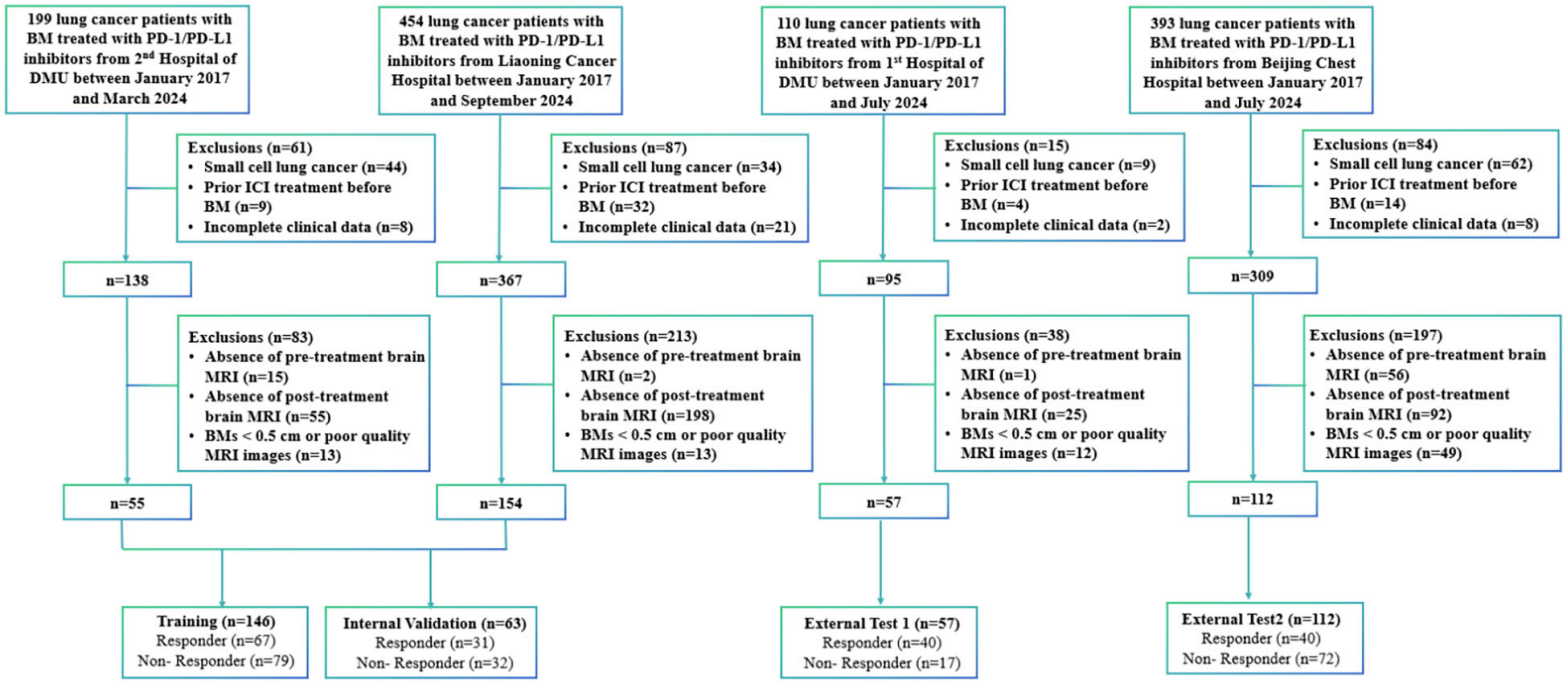
Inclusion and exclusion flowchart for patients.

Participants were included based on: 1) ≥ 18 years old; 2) pathologically established NSCLC; 3) baseline MRI scans performed within 4 weeks prior to PD-1/PD-L1 inhibitors therapy; 4) measurable BMs ≥ 0.5 cm; 5) at least two post-treatment MRI scans to assess treatment response. Exclusion criteria included: 1) a pathological diagnosis of small-cell lung cancer (n=149); 2) previous treatment with PD-1/PD-L1 inhibitors before the diagnosis of BM (n=59); 3) incomplete baseline data or absence of follow-up, clinical, or outcome data (n=39); 4) absence of brain MRI images obtained before or after immunotherapy precluded the assessment of immunotherapy efficacy (n=444); and 5) MRI examinations were excluded due to BMs < 0.5 cm or technically suboptimal image quality that precluded accurate segmentation (n=87). **Figure 2** shows a flowchart of the patient enrolment process.

Clinically relevant variables, including age, gender, smoking status, PD-1/PD-L1 inhibitors regimen, Eastern Cooperative Oncology Group (ECOG) performance status, histologic type of lung cancer, PD-L1 expression in lung cancer tissue, number of metastatic lesions, clinical stage, and laboratory parameters (including routine analysis of blood and tumor markers), were retrieved from the Electronic Medical Record System of each patient within 4 weeks prior to initiation of PD-1/PD-L1 inhibitors therapy. Additionally, commonly used inflammatory and nutritional indices, namely the Prognostic Nutritional Index (PNI) and Systemic Inflammation Response Index (SIRI), were calculated as follows: PNI = serum albumin level (g/L) + 5 × absolute lymphocyte count (×10^9^/L); SIRI = absolute neutrophil count × absolute monocyte count/absolute lymphocyte count.

### 2.2 MRI image processing and treatment response assessment

A comprehensive list of MRI machine parameters from all four participating centers is provided in **Supplementary Material 1**. All MRI images underwent N4 bias field correction using the SimpleITK library to reduce intensity inhomogeneity resulting from scanner-related variations. All volumetric datasets were spatially normalized via 1×1×1 mm isovoxel interpolation to ensure consistent resolution. This preprocessing enhances uniformity in image intensity distribution, supports more reliable feature extraction, and strengthens the robustness of subsequent analyses. The region of interest (ROI) was delineated by two experienced radiologists (NL and XQ) with ITK-SNAP (version 3.8.0) independently. Discrepancies underwent arbitration by a senior neuroradiologist with twenty years’ expertise, guaranteeing ROI selection accuracy and consistency. Intraclass correlation coefficient analysis quantified intra- and inter-observer agreement, considering values ≥ 0.75 indicative of excellent reliability. For multi-lesion cases, analysis prioritized the dominant lesion per established radiological guidelines (12, 13). Independent quantification of target intracranial lesions and comprehensive response assessment to PD-1/PD-L1 inhibitors were performed by respiratory medicine specialists (MD and QW) following the Response Assessment in Neuro-Oncology Brain Metastases criteria.

### 2.3 Habitat generation

A 5×5×5 moving window was used to extract local features from each voxel in the dataset, generating 19 feature vectors per voxel. This window size was chosen to balance the need for capturing adequate spatial context to ensure robust radiomic feature calculation, while preserving anatomical relevance within peritumoral regions (18). Tumor partitioning into phenotypically distinct subregions utilized K-means clustering, where Calinski-Harabasz, Davies-Bouldin, and Silhouette scores determined optimal cluster configuration. To ensure the robustness of habitat definitions against the stochastic nature of K-means initialization, the algorithm was repeated 10 times with different random seeds for each value of k. The cluster assignment with the best overall validation metrics was selected. Detailed methods for habitat generation are provided in **Supplementary Material 2**.

### 2.4 Peritumoral region dilation

**Figure 3:**
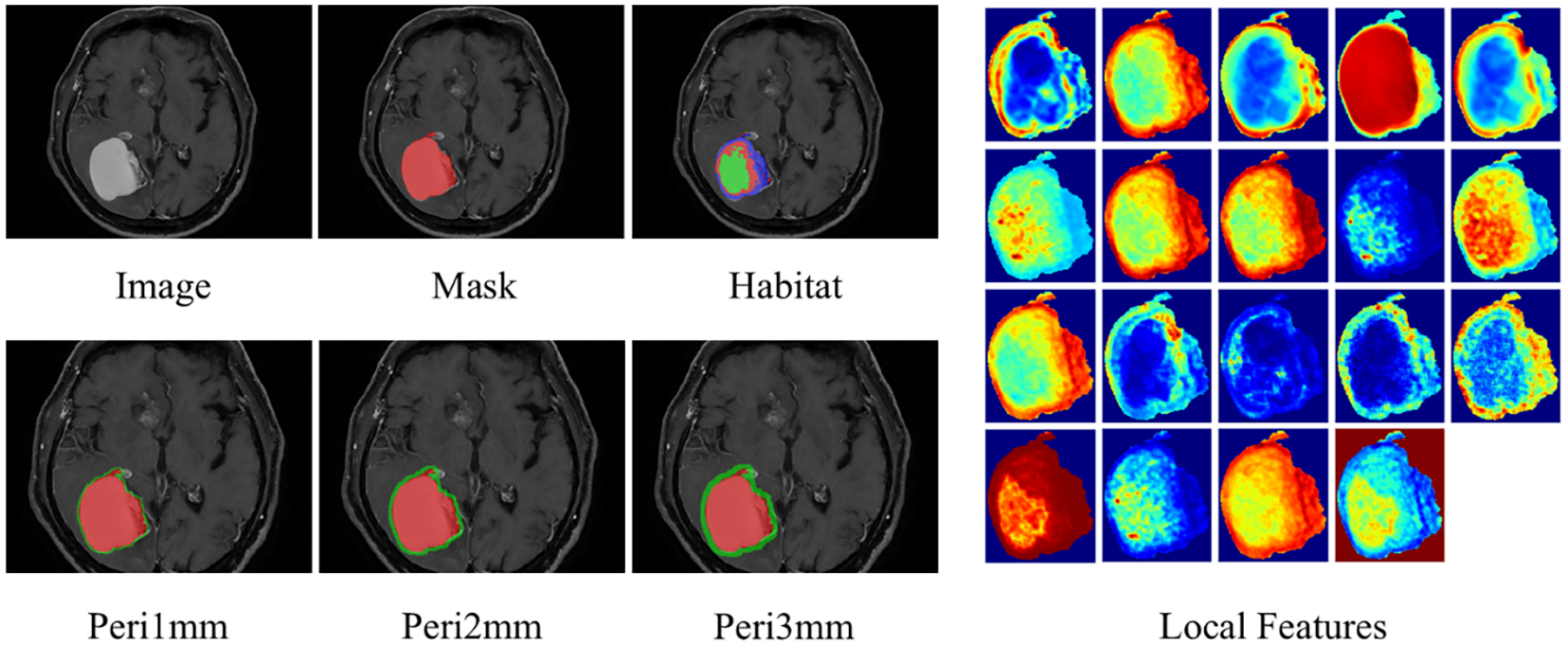
ROI segmentation of brain metastasis, habitat generation, peritumoral region expansion, and local feature display. ROI, region of interest.

Radial dilation of tumor margins (1mm/2mm/3mm) was implemented to quantify peritumoral impact on model efficacy. The radial dilation distances of 1 mm, 2 mm, and 3 mm were selected based on established practices in neuro-oncologic radiomics, where narrow peritumoral margins are often employed to capture the invasive front and immune microenvironment while minimizing the inclusion of distal edema or normal tissue (19, 20). We determined the optimal peritumoral extent through systematic comparison of model performance across different dilation sizes. The habitat and peritumoral regions generated through this process are illustrated in **Figure 3**.

### 2.5 Feature extraction and selection

PyRadiomics 3.0.1 implemented feature extraction compliant with Imaging Biomarker Standardization Initiative (IBSI) guidelines. For intratumoral habitat regions, we performed feature extraction followed by early fusion before modeling. Feature extraction was performed separately for distinct peritumoral margins, followed by construction of individual models. The feature extraction process was consistent across intratumoral, peritumoral, and habitat-based analyses. Radiomic features were systematically categorized into geometric, intensity, and texture domains. Geometric parameters quantified tumor morphology, intensity metrics assessed voxel signal distributions, while textural attributes captured spatial patterns through gray-level concurrence matrix (GLCM), gray-level run length matrix (GLRLM), gray-level size zone matrix (GLSZM), and neighborhood gray-tone difference matrix (NGTDM).

To enhance the generalizability of the model and minimize overfitting, a multi-stage feature selection strategy was adopted. Initially, feature stability and relevance were assessed through statistical methods: independent sample t-tests and Mann-Whitney U tests were applied to identify features with significant differences (p < 0.05) and non-significant associations (p ≥ 0.05), respectively. Subsequently, Pearson correlation coefficients were computed to exclude redundant variables, implementing a 0.9 threshold to mitigate multicollinearity concerns. Further refinement was achieved using a recursive elimination approach to iteratively remove less contributory features. To optimize the feature subset by balancing discriminability and independence, the Minimum Redundancy Maximum Relevance (mRMR) algorithm was employed. The final feature set was refined via Least Absolute Shrinkage and Selection Operator (LASSO) regression, which performed coefficient shrinkage to suppress less informative predictors. Hyperparameter tuning of λ was performed using 10-fold cross-validation, retaining maximally discriminatory features. Learning curves illustrating model performance throughout the selection process are provided in **Supplementary Material 3**.

### 2.6 Model construction

For distinct regions, we developed four radiomic signatures: intratumoral (radiomics), peritumoral 1 mm (Peri-1mm), peritumoral 2 mm (Peri-2mm), and peritumoral 3 mm (Peri-3mm), as well as a habitat subregion (Habitat) model generated through clustering to determine the optimal number of subregions. Clinical risk factors were identified via univariate and multivariate analyses to construct a clinical model. A combined model was subsequently developed by combining clinical factors with intratumoral, optimal peritumoral, and habitat features. Detailed technical descriptions of the model construction process are provided in **Supplementary Material 4**.

### 2.7 Statistical analysis and model performance evaluation

Clinical feature normality was assessed using the Shapiro-Wilk test. Continuous variables were analyzed with parametric (t-test) or nonparametric (Mann-Whitney U test) tests, while categorical variables were evaluated using Chi-square (χ²) tests. Statistical analyses were performed using Statsmodels version 0.13.2, radiomic feature extraction was conducted with PyRadiomics version 3.0.1, and predictive modeling was implemented using Scikit-learn version 1.0.2. Model performance was assessed using the area under the receiver operating characteristic curve (AUC) and its 95% confidence interval (CI), with comparisons made using Delong test. In addition to AUC, other diagnostic metrics, including sensitivity, specificity, positive predictive value (PPV), negative predictive value (NPV), and accuracy, were calculated to evaluate predictive ability across cohorts. Calibration was assessed using calibration curves and Hosmer-Lemeshow (HL) analysis. Clinical net benefit was quantified through decision curve analysis (DCA). The SHapley Additive explanation (SHAP) algorithm was used to interpret the contribution of features, improving model transparency and explaining its impact on predicting intracranial immunotherapy response in NSCLC-BM patients.

## 3 Results

### 3.1 Patients characteristics

Following application of the inclusion criteria, 378 patients were allocated into four cohorts. A combined cohort of 209 patients from the Second Affiliated Hospital of Dalian Medical University (n=55) and Liaoning Cancer Hospital (n=154) was randomly divided in a 7:3 ratio, yielding a training cohort (n=146) and an internal validation cohort (n=63). The performance and fitness of the model were evaluated using the test 1 cohort (n=57) and test 2 cohort (n=112) from the First Affiliated Hospital of Dalian Medical University and Beijing Chest Hospital. All cohort baseline characteristics are comprehensively presented in **Table 1**. **Table 2** presents a comprehensive univariate and multivariate analyses of all clinical features, evaluating their association with response using odds ratios (OR) and the corresponding p-values. It is worth noting that the statistically significant feature, gender, was retained due to its strong predictive ability. **Supplementary Materials 5** compares the performances of various clinical models.

**Table 1:** Baseline characteristic of cohorts.

Characteristics	Training cohort	Internal validation cohort	Test 1 cohort	Test 2 cohort	p value
Age (years)	64.45 ± 7.67	62.12 ± 7.84	65.81 ± 7.96	64.75 ± 9.45	***0.047***
Gender (%)					0.584
Male	97 (66.44)	45 (71.43)	44 (77.19)	75 (66.96)	
Female	49 (33.56)	18 (28.57)	13 (22.81)	37 (33.04)	
Smoking status (%)					0.16
Never	64 (43.84)	35 (55.56)	36 (63.16)	48 (42.86)	
Current/former	82 (56.16)	28 (44.44)	21 (36.84)	64 (57.14)	
Lines of ICIs therapy (%)					1
1-2	110 (75.34)	48 (76.19)	48 (84.21)	77 (68.75)	
>2	36 (24.66)	15 (23.81)	9 (15.79)	35 (31.25)	
Type of ICI (%)					0.567
Anti-PD-1	139 (95.21)	58 (92.06)	45 (78.95)	99 (88.39)	
Anti-PD-L1	7 (4.79)	5 (7.94)	12 (21.05)	13 (11.61)	
Immunotherapy combined with chemotherapy (%)					0.295
No	18 (12.33)	4 (6.35)	8 (14.04)	12 (10.71)	
Yes	128 (87.67)	59 (93.65)	49 (85.96)	100 (89.29)	
ECOG PS (%)					0.895
0-1	68 (46.58)	28 (44.44)	31 (54.39)	93 (83.04)	
≥2	78 (53.42)	35 (55.56)	26 (45.61)	19 (16.96)	
Pathology (%)					0.994
Adenocarcinoma	108 (73.97)	47 (74.60)	39 (68.42)	79 (70.54)	
Squamous	24 (16.44)	10 (15.87)	13 (22.81)	23 (20.54)	
Other	14 (9.59)	6 (9.52)	5 (8.77)	10 (8.93)	
PD-L1 expression in lung cancer tissue (%)					1
≤50	129 (88.36)	55 (87.30)	55 (96.49)	75 (66.96)	
>50	17 (11.64)	8 (12.70)	2 (3.51)	37 (33.04)	
Number of metastatic sites	1.73 ± 1.31	1.62 ± 1.35	1.51 ± 1.28	2.96 ± 1.91	0.509
Number of BM (%)					0.898
Solitary	99 (67.81)	44 (69.84)	36 (63.16)	56 (50.00)	
Multiple	47 (32.19)	19 (30.16)	21 (36.84)	56 (50.00)	
Clinical T stage (%)					0.758
0-2	53 (36.30)	25 (39.68)	12 (21.05)	28 (25.00)	
3-4	93 (63.70)	38 (60.32)	45 (78.95)	84 (75.00)	
Clinical N stage (%)					0.703
0-2	94 (64.38)	43 (68.25)	53 (92.98)	67 (59.82)	
3-4	52 (35.62)	20 (31.75)	4 (7.02)	45 (40.18)	
Clinical M stage (%)					0.878
0-2	19 (13.01)	7 (11.11)	3 (5.26)	8 (7.14)	
3-4	127 (86.99)	56 (88.89)	54 (94.74)	104 (92.86)	
RBC (10^12^/L)	4.29 ± 0.57	4.45 ± 0.59	4.19 ± 0.67	4.18 ± 0.61	0.07
WBC (10^9^/L)	6.83 ± 2.83	6.94 ± 2.42	7.21 ± 4.77	7.62 ± 4.63	0.436
Blood platelet (10^9^/L)	244.83 ± 94.33	246.10 ± 106.53	229.00 ± 93.81	257.75 ± 94.04	0.697
LDH (U/L)	246.26 ± 188.10	241.47 ± 74.12	220.15 ± 67.23	234.15 ± 145.42	0.067
CEA (ng/ml)	53.11 ± 165.98	51.50 ± 122.42	7.20 ± 8.69	39.24 ± 95.81	0.202
CA125 (U/ml)	102.71 ± 607.56	37.91 ± 54.37	29.48 ± 21.55	49.58 ± 93.02	0.577
CA19-9 (U/ml)	29.52 ± 54.86	24.71 ± 40.59	15.43 ± 8.66	29.01 ± 58.03	0.710
PNI	48.87 ± 5.80	49.79 ± 7.49	48.71 ± 3.28	46.06 ± 5.97	0.325
SIRI	1.93 ± 2.02	1.75 ± 1.66	3.03 ± 6.13	2.47 ± 3.81	0.710

ICI, immune checkpoint inhibitor; PD-1, programmed cell death protein 1; PD-L1, programmed death ligand 1; ECOG PS, eastern cooperative oncology group performance status; BM, brain metastasis; RBC, red blood cell; WBC, white blood cell; LDH, lactate dehydrogenase; CEA, carcinoembryonic antigen; CA125, carbohydrate antigen 125; CA19-9, carbohydrate antigen 19-9; PNI, prognostic nutritional index; SIRI, systemic inflammation response index. The bold values denote statistical significance.

**Table 2:** Univariable and multivariable analysis of clinical features.

Feature name	Univariable analysis			Multivariable analysis		
	OR	95% CI	p value	OR	95% CI	p value
Age	1.003	0.996 - 1.011	0.468			
Gender	0.85	0.753 - 0.959	***<0.05***	0.85	0.753-0.959	***<0.05***
Smoking status	1.131	1.009 - 1.267	0.075			
Lines of ICIs therapy	0.858	0.751 - 0.979	0.057			
Type of ICI	0.866	0.677 - 1.107	0.335			
Immunotherapy combined with chemotherapy	1.125	0.933 - 1.355	0.298			
ECOG PS	1.08	0.964 - 1.212	0.266			
Pathology	1.05	0.962 - 1.147	0.36			
PD-L1 expression in lung cancer tissue	0.925	0.775 - 1.103	0.464			
Number of metastatic sites	0.982	0.941 - 1.026	0.5			
Number of BM	1.024	0.905 - 1.158	0.755			
Clinical T stage	1.121	0.996 - 1.261	0.111			
Clinical N stage	0.943	0.836 - 1.064	0.423			
Clinical M stage	1.101	0.926 - 1.310	0.36			
RBC	1.098	0.994 - 1.212	0.121			
WBC	1.018	0.996 - 1.040	0.175			
Blood platelet	1.000	1.000 - 1.001	0.292			
LDH	1.000	1.000 - 1.000	0.774			
CEA (ng/ml)	1.000	1.000 - 1.001	0.439			
CA125 (U/ml)	1.000	1.000 - 1.000	0.517			
CA19-9 (U/ml)	1.000	0.999 - 1.002	0.515			
PNI	1.004	0.995 - 1.013	0.467			
SIRI	0.993	0.964 - 1.022	0.679			

OR, odds ratio; CI, confidence interval; ICI, immune checkpoint inhibitor; ECOG PS, eastern cooperative oncology group performance status; PD-L1, programmed death ligand 1; BM, brain metastases; RBC, red blood cell; WBC, white blood cell; LDH, lactate dehydrogenase; CEA, carcinoembryonic antigen; CA125, carbohydrate antigen 125; CA19-9, carbohydrate antigen 19-9; PNI, prognostic nutritional index; SIRI, systemic inflammation response index. The bold values denote statistical significance.

### 3.2 Habitat clustering and feature selection

**Figure 4:**
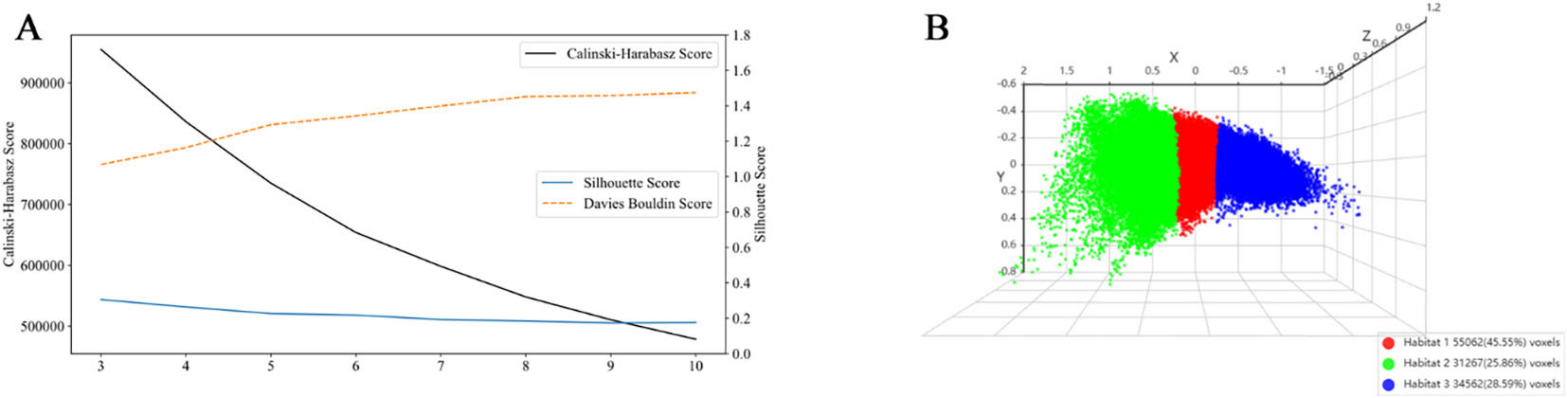
Evaluation of clustering performance and visualization of habitat clusters. **(A)** Cluster validation scores: The Calinski-Harabasz score (black line), Silhouette score (blue line), and Davies-Bouldin score (orange dashed line) are plotted against the number of clusters. At k = 3, the Silhouette score reached its maximum value, indicating that data points were most appropriately assigned to their respective clusters with clear separation from adjacent clusters. The Calinski-Harabasz score also peaked at k = 3, demonstrating an optimal balance between inter-cluster dispersion and intra-cluster cohesion. Conversely, the Davies-Bouldin score attained its minimum value at k = 3, confirming that under this configuration, the clusters were most compact and distinctly separated from one another. All three metrics consistently affirmed that k = 3 provides the best balance between cluster separation and compactness. **(B)** 3D scatter plot showing the three identified habitat clusters, with each cluster color-coded: Habitat 1 (red), Habitat 2 (green), and Habitat 3 (blue). The percentage of voxels belonging to each cluster is indicated in the legend.

We determined the optimal number of clustering centers by systematically evaluating cluster counts ranging from 3 to 10. Based on Calinski-Harabasz, Davies-Bouldin, and Silhouette scores, the optimal number of clusters was determined to be three (**Figure 4**). Robustness analysis conducted through multiple random initializations showed that the voxel assignment consistency exceeded 90% for the k = 3 solution, indicating high algorithmic stability.

For each segmented region, a total of 1,834 handcrafted radiomic features were extracted, covering shape, first-order intensity, and textural characteristics. These included 360 features from first-order statistics, 14 from shape-based descriptors, and the remaining from texture-based methods such as GLCM, GLRLM, GLSZM, and NGTDM. Habitat modeling integrated these features across three clusters, resulting in 5,502 subregional features. Both intratumoral and peritumoral regions contributed 1,834 features each. Feature extraction was performed using Pyradiomics prior to analytical processing. Categorical distributions and dataset structure are illustrated in **Supplementary Materials 6**. Feature selection was conducted using LASSO logistic regression to identify predictors with nonzero coefficients relevant to the Rad-score.

### 3.3 Selection of machine learning algorithms for model construction

Within traditional radiomics models, the ExtraTrees algorithm surpassed counterparts with AUC values of 0.744 (95% CI: 0.623-0.866) in the internal validation cohort, 0.72 (95% CI: 0.554-0.886) in external test 1 cohort, and 0.703 (95% CI: 0.605-0.801) in external test 2 cohort (**Supplementary Materials 7**). For peritumoral analysis, the peri-1 mm RandomForest model exceeded other regional configurations. It achieved an AUC of 0.836 (95% CI: 0.744-0.927) in the internal validation cohort, 0.646 (95% CI: 0.481-0.810) in test 1 cohort, and 0.666 (95% CI: 0.567-0.764) in test 2 cohort (**Supplementary Materials 8**). As for the habitat imaging analysis, in the training cohort, XGBoost achieved the highest AUC of 0.900 (95% CI: 0.852-0.947), outperforming other models: LR (AUC = 0.875; 95% CI: 0.820-0.931), SVM (AUC = 0.867; 95% CI: 0.810-0.924), RandomForest (AUC = 0.844; 95% CI: 0.782-0.906), ExtraTrees (AUC = 0.817; 95% CI: 0.749-0.884), and LightGBM (AUC = 0.866; 95% CI: 0.810-0.923). The superior predictive ability of XGBoost was consistently observed in the internal validation and test cohorts 1 and 2, with AUC values of 0.886 (95% CI: 0.808-0.964), 0.820 (95% CI: 0.712-0.928), and 0.804 (95% CI: 0.725-0.884), respectively, demonstrating its robustness and generalizability (**Supplementary Materials 9**). Based on these results, XGBoost was selected as the optimal model for subsequent analyses due to its consistently high AUC performance across validation and test cohorts.

### 3.4 SHAP analysis

**Figure 5:**
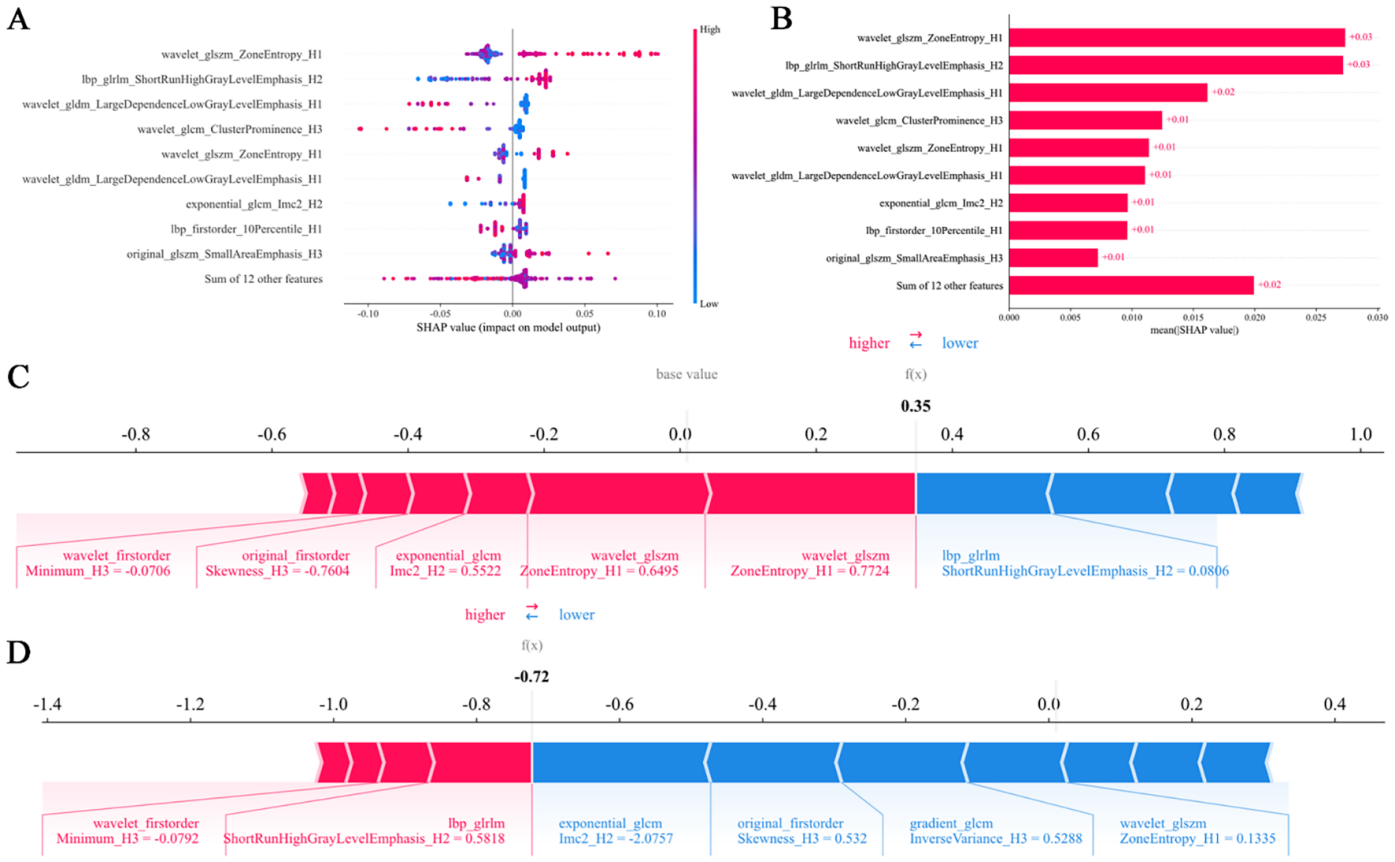
SHAP summary plots **(A, B)** quantify feature contributions to model predictions. Force plots **(C, D)** delineate the decision logic distinguishing responders from non-responders in the habitat imaging model. SHAP, SHapley Additive explanation.

SHAP analysis identified wavelet_glszm_ZoneEntropy_H1 as the most influential feature (**Figure 5A**). Compared to other regions, the H1 region exhibited greater feature importance, highlighting its critical role in enhancing both the predictive accuracy and interpretability of the model (**Figure 5B**). For instance, one patient with a SHAP value of 0.35, above the baseline, was categorized as high-risk. This classification was largely attributable to wavelet_glszm_ZoneEntropy_H1, which contributed positively with a score of 0.7724 toward predicting responsiveness, as indicated by the red arrow in **Figure 5C**. Conversely, another patient with a SHAP value of -0.72, below the baseline, was identified as low-risk. In this case, the feature exponential_glcm_Imc2_H2 exerted a strong negative influence (-2.0757) on the prediction of response, shown by the blue arrow in **Figure 5D**.

### 3.5 Model comparison and evaluation

**Figure 6:**
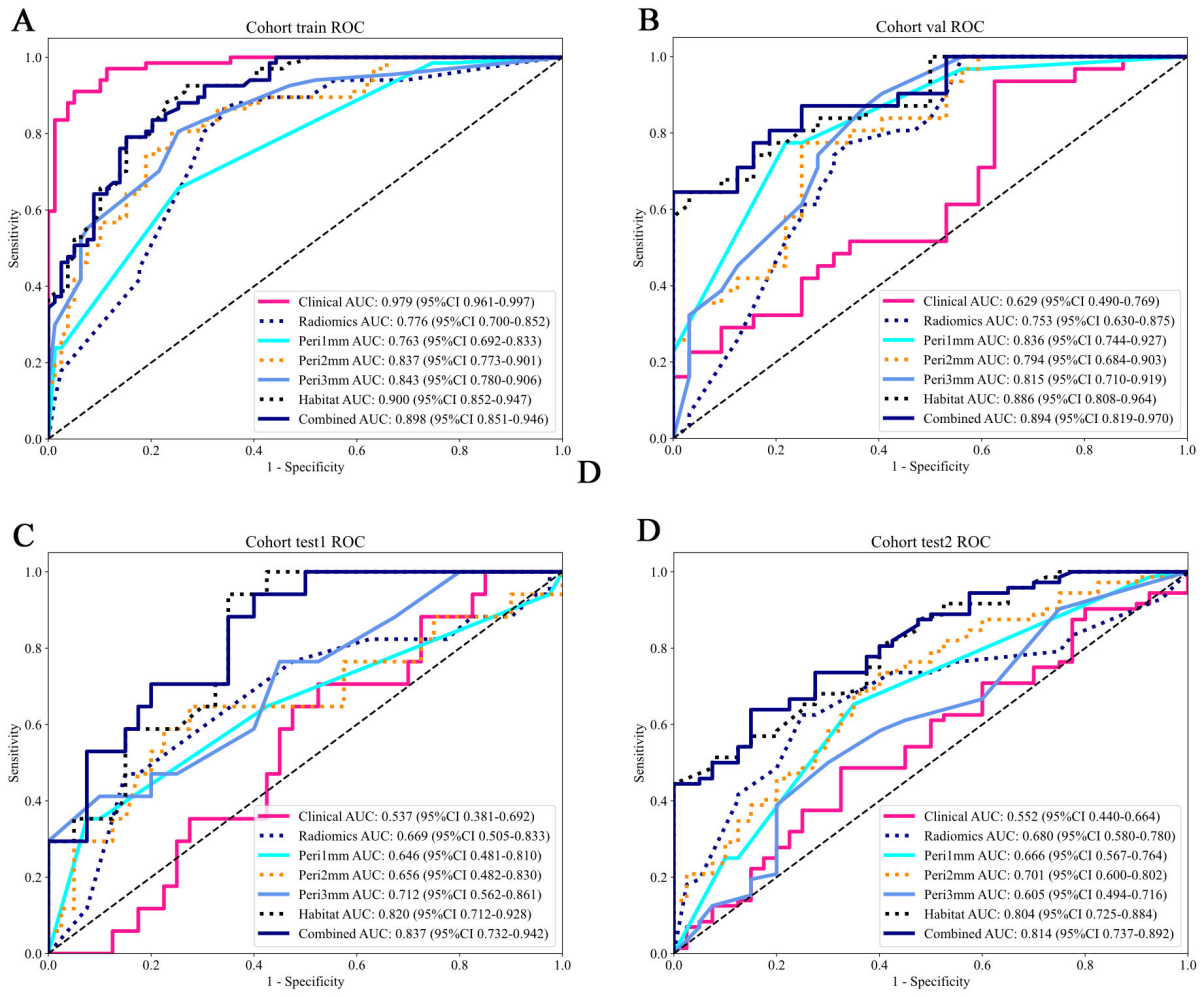
ROC curves of predictive models across different cohorts. The AUC values and their 95% CIs are displayed for each model. The models evaluated include: Clinical (pink), Radiomics (blue), Peritumoral region at 1mm (cyan), Peritumoral region at 2mm (orange), Peritumoral region at 3mm (azure), Habitat (dark blue), and the Combined model (navy blue). The combined model demonstrates higher AUC values across all cohorts, indicating its overall superior performance compared to the individual models. **(A)** Training cohort; **(B)** Internal validation cohort; **(C)** Test 1 cohort; **(D)** Test 2 cohort. ROC, receiver operating characteristic; AUC, area under the curve; CI, confidence interval.

Model predictive performances are comparatively illustrated in **Figure 6** and **Table 3**. Among all the models, the habitat imaging model demonstrated the highest AUC across the internal validation and external test cohorts. In the validation cohort, the habitat model yielded an AUC of 0.886 (95% CI: 0.808-0.964), with sensitivity of 0.613, specificity of 0.969, PPV of 0.950, and NPV of 0.721, outperforming peri-1 mm (AUC = 0.836, 95% CI: 0.744-0.927), peri-2 mm (AUC = 0.794, 95% CI: 0.684-0.903), and peri-3 mm (AUC = 0.815, 95% CI: 0.710-0.919). The combined model, which incorporated habitat features, peri-1 mm, and key clinical variables, attained a slightly higher AUC of 0.894 (95% CI: 0.819-0.970), together with sensitivity of 0.613, specificity of 1.0, PPV of 1.0, and NPV of 0.727, demonstrating the incremental value of multimodal integration. The habitat imaging methodology consistently surpassed comparative models during external testing. Within the test 1 cohort, the habitat model achieved excellent discrimination (AUC = 0.820, 95% CI: 0.712-0.928), with sensitivity of 0.882, specificity of 0.650, PPV of 0.517, and NPV of 0.929, compared with peri-1 mm (AUC = 0.646, 95% CI: 0.481-0.810), peri-2 mm (AUC = 0.656, 95% CI: 0.482-0.830), and peri-3 mm (AUC = 0.712, 95% CI: 0.563-0.861). The combined model further improved performance with an AUC of 0.837 (95% CI: 0.732-0.942), accompanied by sensitivity of 0.882, specificity of 0.600, PPV of 0.484, and NPV of 0.923. Similarly, in the test 2 cohort, the habitat model attained an AUC of 0.804 (95% CI: 0.725-0.884), with specificity of 1.0, PPV of 1.0, sensitivity of 0.431, and NPV of 0.494, while the combined approach yielded a slightly higher AUC of 0.814 (95% CI: 0.737-0.892), with sensitivity of 0.625, specificity of 0.850, PPV of 0.882, and NPV of 0.557, confirming consistent cross-cohort superiority.

**Table 3:** Performance of different models in each cohorts.

Cohort	Signature	Accuracy	AUC	95% CI	Sensitivity	Specificity	PPV	NPV
Training Cohort	Clinical	0.925	0.979	0.961 - 0.997	0.896	0.949	0.937	0.915
	Radiomics	0.747	0.776	0.700 - 0.852	0.851	0.658	0.679	0.839
	Peri-1mm	0.637	0.763	0.692 - 0.833	0.239	0.975	0.889	0.602
	Peri-2mm	0.774	0.837	0.773 - 0.901	0.791	0.759	0.736	0.811
	Peri-3mm	0.747	0.843	0.780 - 0.906	0.701	0.785	0.734	0.756
	Habitat	0.815	0.900	0.852 - 0.947	0.910	0.734	0.744	0.906
	Combined	0.815	0.898	0.851 - 0.946	0.776	0.848	0.812	0.817
Internal validation Cohort	Clinical	0.635	0.629	0.490 - 0.769	0.903	0.375	0.583	0.800
	Radiomics	0.683	0.753	0.630 - 0.875	0.903	0.469	0.622	0.833
	Peri-1mm	0.619	0.836	0.744 - 0.927	0.226	1	1	0.571
	Peri-2mm	0.746	0.794	0.684 - 0.903	0.742	0.75	0.742	0.750
	Peri-3mm	0.746	0.815	0.710 - 0.919	0.871	0.625	0.692	0.833
	Habitat	0.794	0.886	0.808 - 0.964	0.613	0.969	0.95	0.721
	Combined	0.810	0.894	0.819 - 0.970	0.613	1	1	0.727
Test 1 Cohort	Clinical	0.526	0.537	0.382 - 0.692	0.647	0.475	0.344	0.760
	Radiomics	0.684	0.669	0.505 - 0.833	0.118	0.925	0.4	0.712
	Peri-1mm	0.702	0.646	0.481 - 0.810	0	1	0	0.702
	Peri-2mm	0.684	0.656	0.482 - 0.830	0.588	0.725	0.476	0.806
	Peri-3mm	0.596	0.712	0.563 - 0.861	0.588	0.6	0.385	0.774
	Habitat	0.719	0.820	0.712 - 0.928	0.882	0.65	0.517	0.929
	Combined	0.684	0.837	0.732 - 0.942	0.882	0.6	0.484	0.923
Test 2 Cohort	Clinical	0.545	0.552	0.440 - 0.664	0.472	0.675	0.723	0.415
	Radiomics	0.598	0.680	0.580 - 0.780	0.486	0.800	0.814	0.464
	Peri-1mm	0.473	0.666	0.568 - 0.765	0.250	0.875	0.783	0.393
	Peri-2mm	0.679	0.701	0.600 - 0.802	0.722	0.600	0.765	0.545
	Peri-3mm	0.536	0.605	0.494 - 0.716	0.389	0.800	0.778	0.421
	Habitat	0.634	0.804	0.725 - 0.884	0.431	1	1	0.494
	Combined	0.705	0.814	0.737 - 0.892	0.625	0.850	0.882	0.557

AUC, area under the curve; CI, confidence interval; PPV, positive predictive value; NPV, negative predictive value.

**Figure 7:**
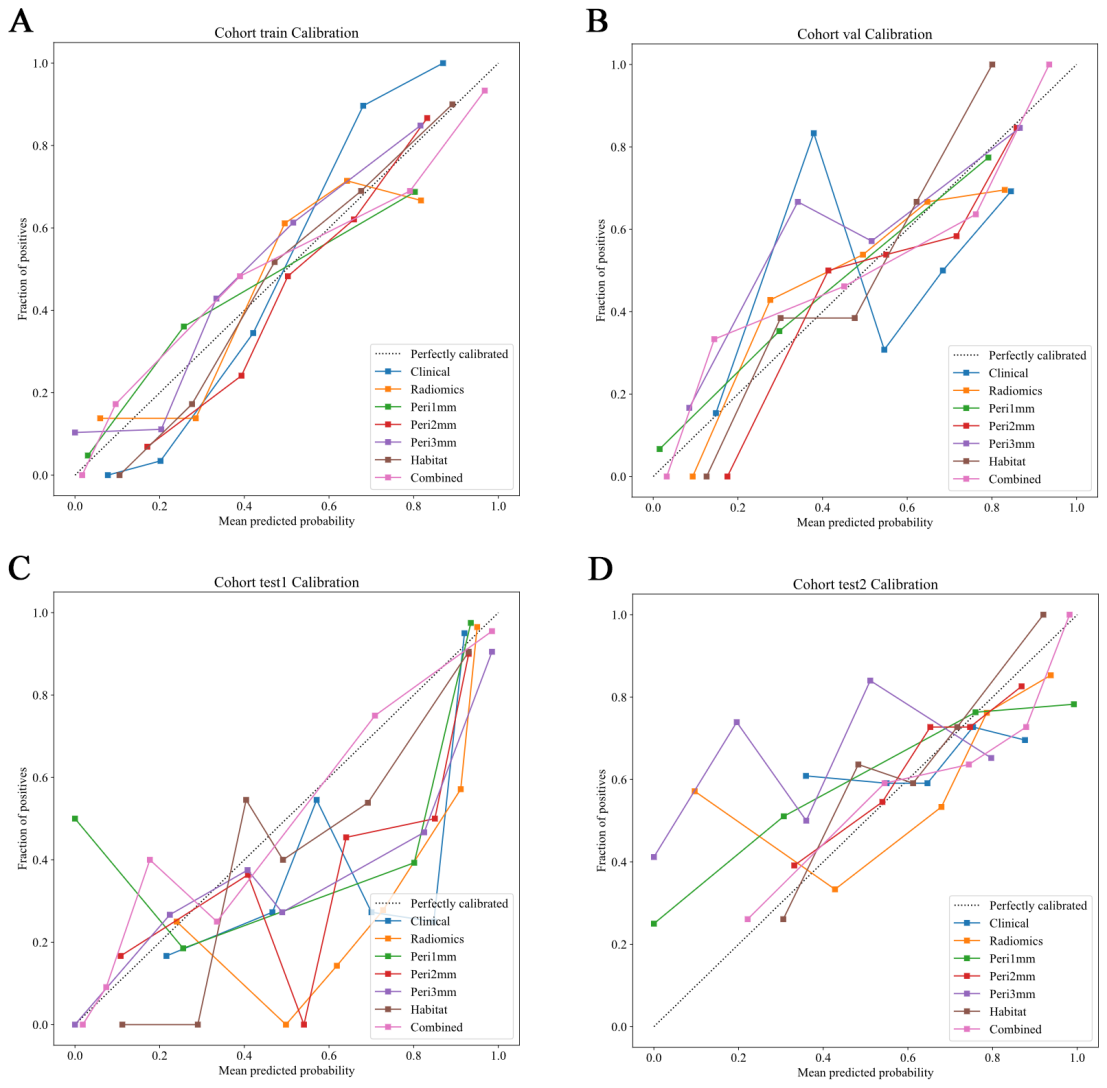
Calibration curves of predictive models across different cohorts. Calibration curves comparing the predicted probabilities and the fraction of positives for various predictive models in different cohorts. The dotted line represents perfect calibration, where predicted probabilities align exactly with observed frequencies. The models evaluated include: Clinical (blue), Radiomics (orange), Peritumoral region at 1mm (green), Peritumoral region at 2mm (red), Peritumoral region at 3mm (purple), Habitat (brown), and the Combined model (pink). The calibration curves show how well the models predict the fraction of positive cases, with better-calibrated models closer to the dotted line. The combined model generally demonstrates superior calibration across all cohorts. **(A)** Training cohort; **(B)** Internal validation cohort; **(C)** Test 1 cohort; **(D)** Test 2 cohort.

**Figure 8:**
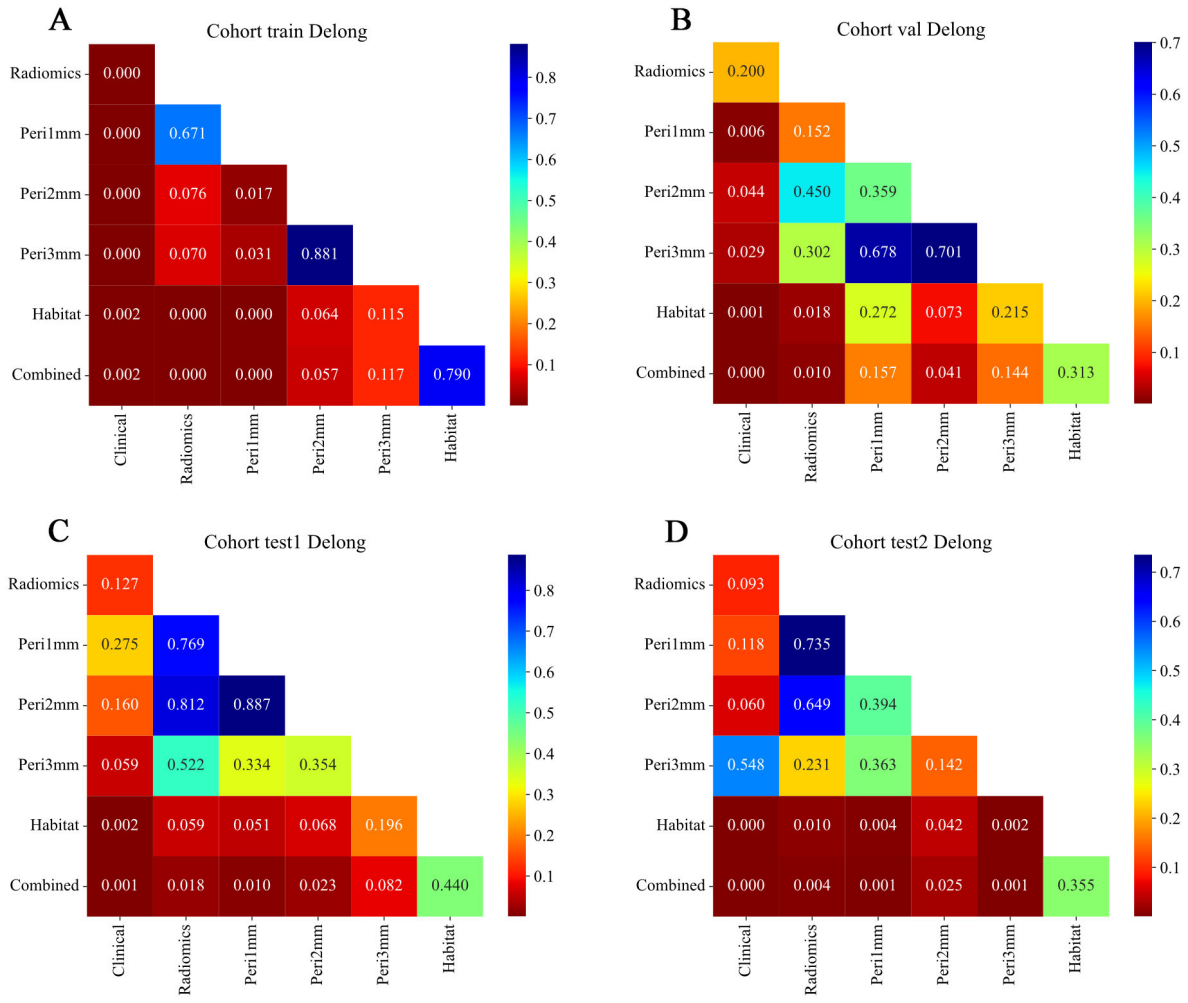
Delong test results comparing model performance across different feature sets and cohorts. Heatmaps showing pairwise p-values from Delong tests for ROC curves of models based on different feature types: Radiomics, Peritumoral region at 1mm, Peritumoral region at 2mm, Peritumoral region at 3mm, Habitat, and Combined. **(A)** Training cohort; **(B)** Internal validation cohort; **(C)** Test 1 cohort; **(D)** Test 2 cohort. The color scale represents p-values, with lower values (red) indicating statistically significant differences between model performances and higher values (blue) indicating non-significant differences. Diagonal elements correspond to self-comparisons and are set to the maximum p-value for visual consistency. Combined models generally demonstrate significant improvement over individual feature-based models across cohorts. ROC, receiver operating characteristic.

**Figure 9:**
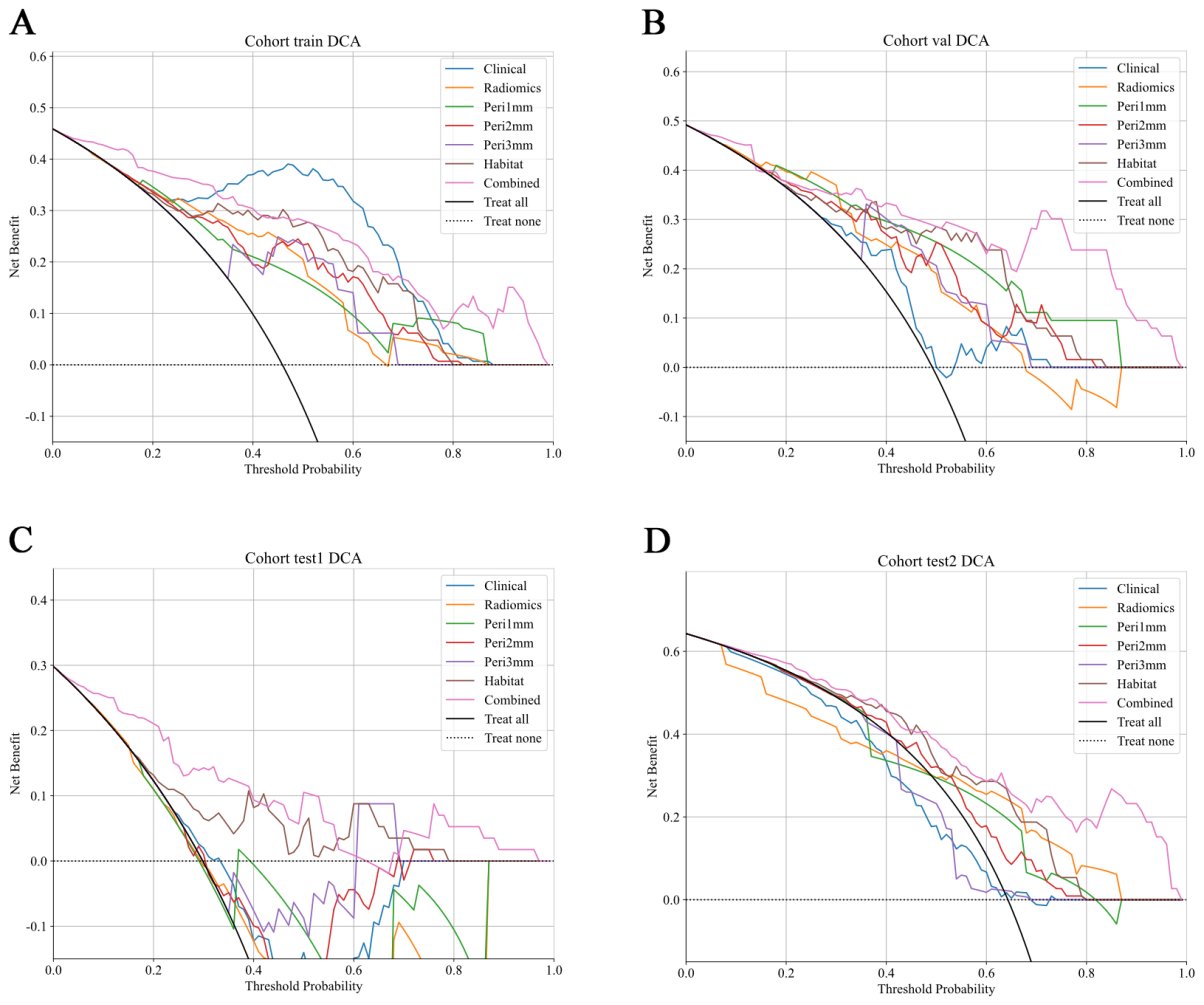
DCA of predictive models across different cohorts. DCA curves show the net clinical benefit of various predictive models at different threshold probabilities in different cohorts. The models evaluated include: Clinical (blue), Radiomics (orange), Peritumoral region at 1mm (green), Peritumoral region at 2mm (red), Peritumoral region at 3mm (purple), Habitat (brown), and the Combined model (pink). These curves represent the net clinical benefit, with the x-axis showing the threshold probability for treatment, and the y-axis showing the net benefit. The dashed black line represents the scenario where no treatment is applied, while the solid black line represents the scenario where all patients are treated. The combined model generally demonstrates a higher net benefit at all threshold probabilities compared to other models, highlighting its clinical value across all cohorts. **(A)** Training cohort; **(B)** Internal validation cohort; **(C)** Test 1 cohort; **(D)** Test 2 cohort. DCA, decision curve analysis.

**Figure 10:**
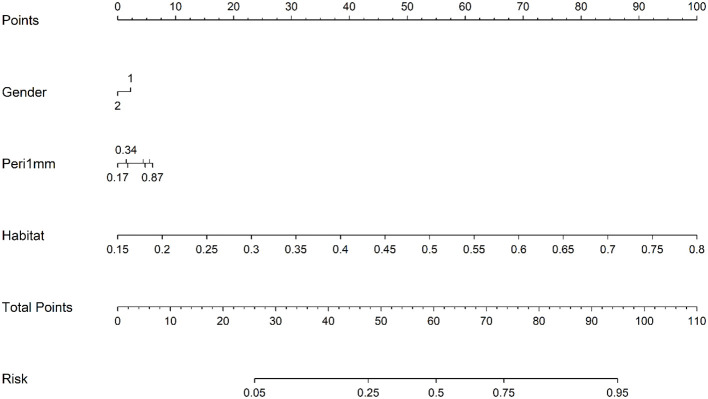
Prognostic nomogram integrating clinical variables, peri-1mm imaging characteristics, and habitat signatures for predicting intracranial therapeutic response.

The HL test evaluates model calibration by comparing predicted probabilities with observed outcomes, where lower values indicate better calibration. Our combined model showed strong calibration, with HL values of 0.326, 0.069, 0.897, and 0.965 in the training, internal validation, test 1, and test 2 cohorts, respectively. This highlights its accuracy and reliability (**Figure 7**). **Figure 8** compares the significance of improvement among the different signatures across the datasets. The Delong test demonstrated a statistically superior predictive performance of the combined model compared to the comparators (P < 0.05). **Figure 9** depicts DCA curves for training and testing cohorts, revealing significantly enhanced net clinical benefit from the combined model’s predictions. Additionally, **Figure 10** presents a nomogram visualizing the combined model’s output.

## 4 Discussion

Precise prediction of intracranial response to PD-1/PD-L1 inhibitors in NSCLC-BM patients is critical for personalized immunotherapy and improved survival outcomes. This study developed a novel approach that integrates habitat imaging and peritumoral radiomics signatures to comprehensively characterize imaging information with the aim of accurately predicting intracranial immunotherapy outcomes in NSCLC-BM cohorts. The habitat imaging model demonstrated superior intracranial efficacy prediction relative to comparator radiomic models, concurrently achieving peak clinical net benefit. The integrated combined model, incorporating radiomic signatures, habitat imaging features, peri-1 mm features, and clinical factors, demonstrated exceptional discriminatory capability and satisfactory calibration across cohorts.

While recent studies have reported intracranial responses to PD-1/PD-L1 inhibitors in patients with NSCLC with BMs, predicting their efficacy against BM remains a significant challenge (21–23). Following the emergence of MRI-based radiomics, multiple studies have employed this approach to predict responses to intracranial immunotherapy in BM cohorts. Shi et al. (12) constructed and clinically validated an MRI-derived radiomic nomogram for 101 small-cell lung cancer patients with BMs receiving ICIs treatment. Their model, incorporating a radiomics score and clinical factors, achieved a validation cohort AUC of 0.875 (95% CI: 0.754–0.996). Xu et al. (13) established a clinical-radiomic model using baseline MRI data from 174 ICI-treated NSCLC-BM patients, achieving validated discrimination (AUC = 0.833; 95%CI: 0.720–0.946). Nevertheless, these investigations characteristically treated tumors as homogeneous entities, focusing exclusively on radiomic feature extraction from the entire tumor volume. Research has indicated that tumors, particularly those with BM originating from NSCLC, exhibit a complex immune microenvironment characterized by significant spatial heterogeneity, which contains information relevant to tumor progression and the efficacy of immunotherapy (24–26). Consequently, the approaches in the aforementioned studies may have overlooked intratumoral spatial heterogeneity and failed to capture potentially critical imaging information relevant to immunotherapy efficacy assessments.

To address these limitations, our study introduces a habitat imaging model designed to systematically identify, analyze, and quantify tumor heterogeneity. Recently, Yang et al. (27) implemented a habitat imaging radiomics framework with preoperative cranial MRI to predict EGFR exon 19/21 mutations in patients with NSCLC-BM, which demonstrated excellent predictive performance. Based on this finding, our model may also holds potential for predicting intracranial responses to immunotherapy in patients with NSCLC-BM. In this research, the model employed unsupervised K-means clustering to delineate BM subregions and identify three distinct habitats, which is consistent with studies reporting optimal predictive reliability from limited subregions (28, 29). Implementation of habitat-based radiomic extraction markedly improved prognostic capability, with resultant AUCs reaching 0.886 in internal validation, 0.82 in test 1 cohort, and 0.804 in test 2 cohort. Due to the highly invasive nature of tissue sampling for BMs, our radiomic analysis was conducted on imaging features and was not directly correlated with histopathological or molecular results (30). Notably, the implementation of SHAP value analysis enhanced the interpretability of the model and identified wavelet features based on textures and H1 subregions as key predictors. The SHAP analysis highlighted the wavelet-based texture features as major contributors to the model’s predictive performance. The features likely reflect underlying pathophysiological processes within the tumor microenvironment. For instance, a high value of wavelet_glszm_ZoneEntropy_H1, which captures heterogeneity in zone size and intensity distribution across multiple scales, may indicate the presence of necrotic tissue areas or heterogeneous immune cell infiltration, both known to influence immunotherapy response (31). The dominance of H1-derived features suggests that this subregion may represent a biologically distinct habitat, potentially characterized by high cellular density, vascular abnormalities, or immune exclusion, which could influence drug delivery and immune cell trafficking (32, 33). By inferring such associations between radiomic features and their pathological bases, our model may offer a non-invasive means to probe tumor microenvironmental states that may affect response to PD-1/PD-L1 inhibitors.

The peritumoral interface, representing the transitional zone between neoplastic tissue and adjacent normal structures, significantly mediates drug delivery through dynamic remodeling of immune cell distribution, vascular networks, and extracellular matrix composition (34). Systematic evaluation of this region within millimeter-scale radial distances will provide evidence linking the peritumoral zone to treatment outcomes, consistent with the current understanding of tumor-stromal interactions (35, 36). In a previous study, Han et al. (37) previously formulated CT-based predictive frameworks fusing intratumoral and peritumoral characteristics to forecast major pathological responses after neoadjuvant chemoimmunotherapy in NSCLC patients. Integration of tumoral and peritumoral features exceeded solitary intratumoral analysis in predictive capability, yielding a validated AUC of 0.831 (0.7255-0.9360). Our findings demonstrate that the peritumoral zone, particularly within 1 mm of the tumor margin, plays a significant role in predicting the response to PD-1/PD-L1 inhibitors in patients with NSCLC-BM. This may be due to its potential as an immunologically active niche enriched with cytotoxic T cells or tertiary lymphoid structures (38). This underscores the central role of the tumor microenvironment in tumor biology and therapeutic outcomes, as immunotherapy efficacy critically depends on the complex interplay among tumor cells, stromal components, immune infiltrates, and vasculature (39).

Although PD-L1 expression is an established biomarker of immunotherapy in NSCLC, its ability to predict intracranial outcomes in patients with BM remains unclear (40). In our study cohort, PD-L1 expression did not emerge as a significant predictor in multivariable analysis, underscoring the potential complementary value of non-invasive radiomics biomarkers in capturing tumor heterogeneity that may be missed by single-site biopsies or static blood-based assays. Similarly, although TMB was not routinely available in our multi-center cohort, future studies directly integrating radiomics features with TMB and PD-L1 data may yield a more comprehensive predictive framework. Such a multimodal approach aligns with the evolving paradigm of personalized oncology, as illustrated by other predictive models for brain metastasis risk, such as the algorithm developed by Armocida et al. for predicting postoperative brain metastasis development (41).Notably, our combined model integrating habitat, peri-1 mm, and significant clinical features significantly improved the performance, highlighting the synergistic value of multimodal integration. This integration further confirms that combining imaging features with clinical data can create a more robust and generalizable model for predicting treatment responses (42, 43). From a clinical perspective, our combined model demonstrated robust calibration effects and net benefits, supporting its potential as a decision-support tool. By categorizing patients into responders and non-responders, this model can help tailor personalized immunotherapy regimens for patients with NSCLC-BM, thereby reducing ineffective treatments and ICI-associated toxicities.

Although our habitat-peritumoral radiomics model showed promising performance, it is important to stress the exploratory nature of these findings and the limitations of this study. First, the retrospective approach carries inherent selection bias risks. Second, our radiomic analysis focused on the dominant lesion in patients with multiple BMs. While this approach is methodologically consistent and clinically practical, it inherently cannot capture the full spectrum of inter-metastatic heterogeneity. Future studies may consider comprehensive multi-lesion profiling strategies to address this complexity and better characterize heterogeneity across metastases. Third, while this study indicated that habitat-based MRI radiomics analysis can predict the immunotherapy response in patients with NSCLC and BM, the current findings are based solely on imaging biomarkers without histopathological confirmation, due to the challenges in tissue sampling. Thus, future prospective trials are necessary to evaluate the clinical utility and generalizability of this approach.

## 5 Conclusion

From a clinical perspective, our combined model demonstrated strong calibration and net benefit for predicting intracranial response to PD-1/PD-L1 inhibitors in NSCLC patients with BMs in this retrospective analysis, supporting its potential as a decision-support tool to help clinicians tailor personalized immunotherapy regimens, thereby reducing ineffective treatments and immune-related toxicities. As this model was developed in an exploratory retrospective study, prospective validation remains necessary.

## Data Availability

The original contributions presented in the study are included in the article/**Supplementary Material**. Further inquiries can be directed to the corresponding authors.
