# Supplementary material for: Prediction of intracranial response to PD-1/PD-L1 inhibitors therapy in brain metastases originating from non-small cell lung cancer using habitat imaging and peritumoral radiomics: a multicenter study
Source: Front Oncol. 2025 Oct 28;15:1657290. doi: 10.3389/fonc.2025.1657290 (PMC12602230; doi:10.3389/fonc.2025.1657290)
Supplement: Supplementary file 1 [file DataSheet1.pdf]

Supplementary Material

1. Parameters of MRI machines in each center

Center 1- The Second Affiliated Hospital of Dalian Medical University
Patients were scanned with a 3.0-T MRI scanner (Siemens Verio).The contrast agent was gadolinium diethylenetriaminepentaacetic acid (Gd-DTPA, Bayer, Berlin, Germany). After the contrast agent injection with the dose of 0.2 mL/kg and injection speed of 2 mL/s, T1CE MR images were obtained. The scanning parameters of T1CE MRI were as follows: repetition time [TR] = 1800 ms, echo time [TE] = 9 ms, matrix size = 320 × 320, field of view [FOV] = 240 × 192 mm, and slice thickness = 5 mm.

Center 2- Liaoning Cancer Hospital
Patients were scanned with a 3.0-T MRI scanner (Philips Ingenia). The contrast agent was gadolinium diethylenetriaminepentaacetic acid (Gd-DTPA, Bayer, Berlin, Germany). After the contrast agent injection with the dose of 0.2 mL/kg and injection speed of 3 mL/s, T1CE MR images were obtained. The scanning parameters of T1CE MRI were as follows: TR = 260 ms, TE = 4.61 ms, matrix size = 512 × 512, FOV = 230 × 230 mm, and slice thickness = 5 mm.

Center 3- The First Affiliated Hospital of Dalian Medical University
Patients were scanned with a 3.0-T MRI scanner (Philips Achieva). The contrast agent was gadolinium diethylenetriaminepentaacetic acid (Gd-DTPA, Bayer, Berlin, Germany). After the contrast agent injection with the dose of 0.2 mL/kg and injection speed of 3 mL/s, T1CE MR images were obtained. The scanning parameters of T1CE MRI were as follows: TR = 600 ms, TE = 28 ms, matrix size = 864 × 864, FOV = 240 × 240 mm, and slice thickness = 5 mm.

Center 4- Beijing Chest Hospital
Patients were scanned with a 3.0-T MRI scanner (SIGAL Pioneer). The contrast agent was gadolinium diethylenetriaminepentaacetic acid (Gd-DTPA, Bayer, Berlin, Germany). Following the intravenous administration of a gadolinium-based contrast agent at a dose of 0.1 mmol per kilogram of body weight, T1CE MR images were acquired. The scanning parameters of T1CE MRI were as follows: TR = 250 ms, TE = 2.46 ms, matrix size = 256 × 256, FOV = 240 × 240 mm, and slice thickness = 5 mm.

2. Habitat generation process

Our methodology for delineating tumor habitat regions was multifaceted and involved several complex steps:

- **Comprehensive Radiomic Feature Extraction:** This process involved extracting detailed local features from each voxel in the dataset using a $5 \times 5 \times 5$ moving window. These features encompass a variety of measurements and attributes, including intensity, texture, and other statistical properties, which are crucial for understanding the intricate details of the dataset. Such detailed insights enable more precise modeling and analysis.

  In this study, 19 radiomic features were extracted from each voxel, offering a multidimensional characterization of each subregion. These features included a range of shape descriptors, textural features, and first-order statistical attributes. The specific features extracted were: firstorder_Entropy, firstorder_MeanAbsoluteDeviation, firstorder_Median, glcm_DifferenceAverage, glcm_DifferenceEntropy, glcm_DifferenceVariance, glcm_Imc1, glcm_Imc2, glcm_InverseVariance, glcm_JointEnergy, glcm_JointEntropy, glcm_SumEntropy, glrlm_LongRunEmphasis, glrlm_RunEntropy, glrlm_RunVariance, glszm_SizeZoneNonUniformityNormalized, glszm_SmallAreaHighGrayLevelEmphasis, ngtdm_Contrast, and ngtdm_Strength.

Entropy: Entropy specifies the uncertainty/randomness in the image values.

$$entropy = -\sum_{i=1}^{N_g} p(i)\log_2(p(i)+\epsilon)$$

Mean Absolute Deviation (MAD) : MAD is the mean distance of all intensity values from the Mean Value of the image array.

$$MAD = \frac{1}{N_p}\sum_{i=1}^{N_p} |X(i) - \bar{X}|$$

Difference Entropy: Measures the randomness/variability in neighborhood intensity value differences.

$$difference\_entropy = \sum_{k=0}^{N_g-1} p_{x-y}(k)\log_2\left(p_{x-y}(k)+\epsilon\right)$$

Difference Variance: A measure of heterogeneity, giving higher weights to differing intensity level pairs.

$$difference\_variance = \sum_{k=0}^{N_g-1} (k-DA)^2 p_{x-y}(k)$$

Joint Energy: A measure of homogeneous patterns in the image.

$$joint\_energy = \sum_{i=1}^{N_g}\sum_{j=1}^{N_g} \left(p(i,j)\right)^2$$

Joint Entropy: Measures the randomness/variability in neighborhood intensity values.

$$joint\_entropy = -\sum_{i=1}^{N_g}\sum_{j=1}^{N_g} p(i,j)\log_2(p(i,j)+\epsilon)$$

- **K-means Subregion Clustering:** The K-means algorithm was employed to analyze the multidimensional feature space derived from the radiomic features. This method clustered all voxels and their associated characteristics, exploring a variety of cluster centers, ranging from 3 to 10, to categorize distinct habitat regions within the tumor.
  The K-means algorithm functions by partitioning data into K distinct clusters. It iteratively updates the centroids of these clusters to minimize the sum of squares within each cluster. The central component of the K-means algorithm is the objective function, which is optimized to achieve effective clustering.

$$J = \sum_{i=1}^{N}\sum_{k=1}^{K} w_{ik} \times \| x_i - \mu_k \|^2$$

  - J is the objective function.
  - N is the number of data points.
  - K is the number of clusters.
  - w_{ik} is a binary indicator (1 if data point i is in cluster k, 0 otherwise).
  - x_i is the ith data point.
  - μ_k is the centroid of cluster k.
  - $\| x_i - \mu_k \|^2$ is the squared Euclidean distance between data point i and centroid k.

- **Habitat Region Synthesis:** Following the clustering analysis, subregions with identical cluster IDs were amalgamated. This synthesis resulted in the formation of comprehensive habitat regions, each representing a unique microenvironmental characteristic within the tumor.

3. Learning curves

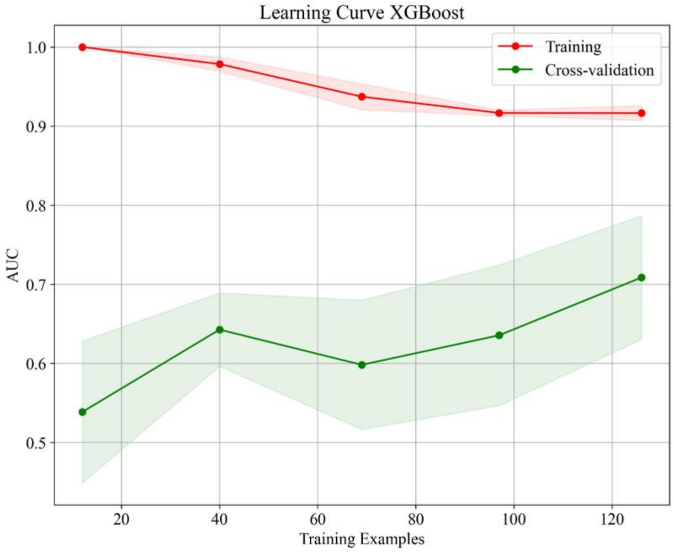

Supplementary Figure 1. Learning curves of the study. The learning curve analysis showed that training performance decreased while cross-validation performance increased and stabilized as the sample size grew. With larger training cohorts, the two curves gradually converged, indicating reduced overfitting and improved model generalizability.

4. Details regarding the construction of the models

- **Radiomics:** Extracted intratumoral features were modeled via logistic regression, support vector machines (SVM), RandomForest, ExtraTrees, XGBoost, and LightGBM algorithms, capitalizing on both linear and nonlinear decision boundaries to augment discriminatory capacity. The selection of all models was based on their performance on the validation set, with the best-performing model chosen for signature comparison.
- **Habitat imaging model:** Features from the clustered tumor subregions were fused before modeling. This approach preserved the spatial heterogeneity of tumor habitats, thereby enabling robust prediction of the immunotherapy response in patients with NSCLC-BM.
- **Peri-X mm imaging model:** Features extracted from each peritumoral region were modeled independently. To guarantee cross-model comparability, the established radiomics framework's feature selection methodology and machine learning algorithms were uniformly applied.
- **Clinical model:** All available clinical features were incorporated to construct a clinical model. For missing values, median imputation was applied to categorical variables, while continuous variables were imputed using the mean value. Clinical models were constructed leveraging stepwise-selected features from univariate and multivariate logistic regression analyses.
- **Combined model:** To develop a comprehensive predictive model, a multivariate stepwise selection process was performed. Feature integration combined clinical characteristics with peritumoral and habitat profiles, selecting only variables meeting the univariate significance criterion ($p < 0.05$). This integrative approach enhanced predictive performance by leveraging complementary information from tumor morphology, spatial heterogeneity, and clinical parameters.

5. Details of clinical model

Supplementary Table 1. Performance of machine learning algorithms for clinical model in each cohorts

Machine learning algorithms	Accuracy	AUC	95% CI	Sensitivity	Specificity	PPV	NPV	Cohort
LR	0.774	0.855	0.796 - 0.915	0.776	0.772	0.743	0.803	Train
LR	0.524	0.503	0.357 - 0.649	0.516	0.531	0.516	0.531	Internal validation
LR	0.474	0.485	0.319 - 0.652	0.588	0.425	0.303	0.708	Test 1
LR	0.527	0.555	0.440 - 0.670	0.528	0.525	0.667	0.382	Test 2
SVM	0.582	0.711	0.626 - 0.795	0.806	0.392	0.529	0.705	Train
SVM	0.571	0.518	0.372 - 0.664	0.774	0.375	0.545	0.632	Internal validation
SVM	0.456	0.716	0.562 - 0.871	0.882	0.275	0.341	0.846	Test 1
SVM	0.625	0.587	0.472 - 0.702	0.764	0.375	0.687	0.469	Test 2
RandomForest	0.911	0.979	0.961 - 0.997	0.925	0.899	0.886	0.934	Train
RandomForest	0.508	0.629	0.490 - 0.769	0.548	0.469	0.5	0.517	Internal validation
RandomForest	0.561	0.537	0.381 - 0.692	0.647	0.525	0.367	0.778	Test 1
RandomForest	0.536	0.552	0.440 - 0.664	0.528	0.55	0.679	0.393	Test 2
ExtraTrees	0.616	0.677	0.590 - 0.764	0.567	0.658	0.585	0.642	Train
ExtraTrees	0.603	0.558	0.410 - 0.707	0.839	0.375	0.565	0.706	Internal validation
ExtraTrees	0.649	0.578	0.421 - 0.735	0.412	0.75	0.412	0.75	Test 1
ExtraTrees	0.518	0.523	0.413 - 0.633	0.458	0.625	0.687	0.391	Test 2
XGBoost	0.76	0.812	0.743 - 0.880	0.746	0.772	0.735	0.782	Train
XGBoost	0.603	0.601	0.459 - 0.742	0.548	0.656	0.607	0.6	Internal validation
XGBoost	0.509	0.516	0.354 - 0.678	0.471	0.525	0.296	0.7	Test 1
XGBoost	0.464	0.471	0.363 - 0.578	0.389	0.6	0.636	0.353	Test 2
LightGBM	0.678	0.744	0.666 - 0.822	0.806	0.57	0.614	0.776	Train
LightGBM	0.54	0.611	0.480 - 0.743	0.71	0.375	0.524	0.571	Internal validation
LightGBM	0.561	0.531	0.374 - 0.688	0.412	0.625	0.318	0.714	Test 1
LightGBM	0.571	0.483	0.371 - 0.595	0.722	0.3	0.65	0.375	Test 2

Abbreviation: AUC: area under the curve; CI: confidence interval; PPV: positive predictive value; NPV: negative predictive value; LR, logistic regression; SVM, support vector machine.

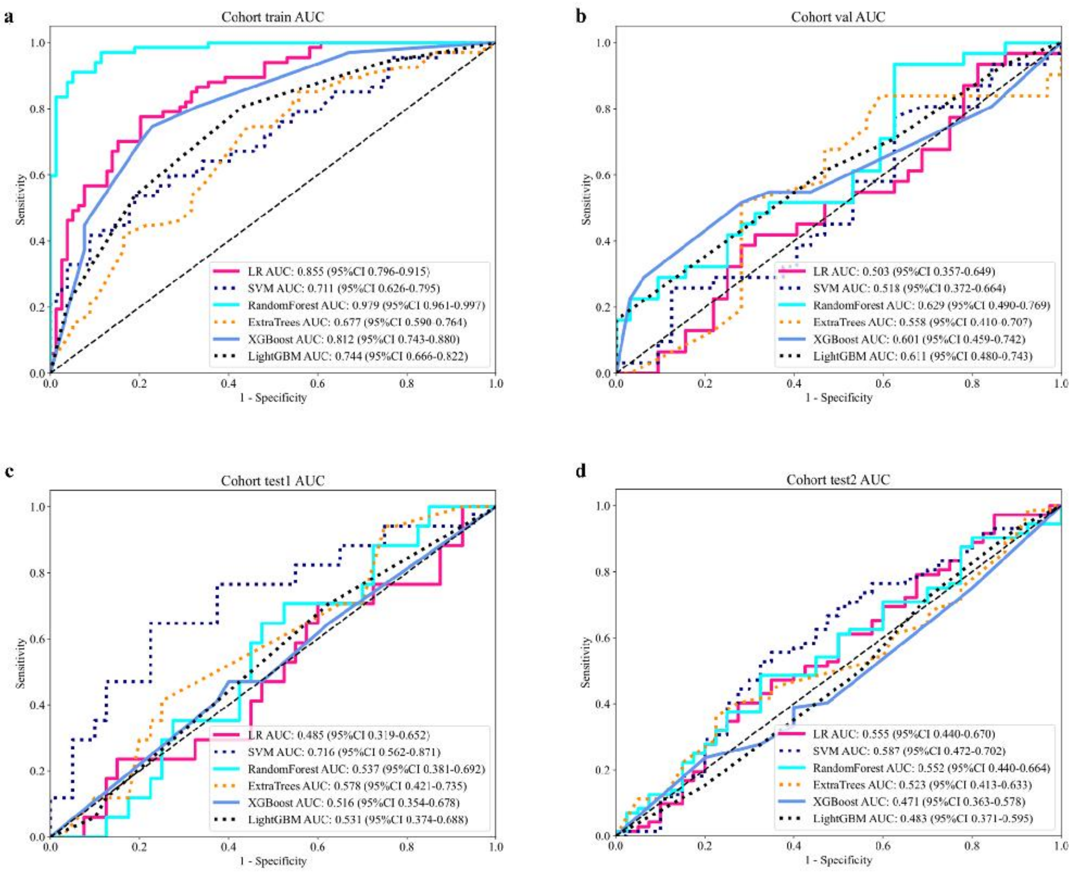

Supplementary Figure 2. ROC curves of multiple machine learning algorithms of clinical model across four independent cohorts. The figure illustrates the discriminatory performance of six distinct classifiers, with AUC values and corresponding 95% CIs provided for each model. The algorithms evaluated include: LR, SVM, RandomForest, Extra Trees, XGBoost, and LightGBM. (a) Training cohort; (b) Internal validation cohort; (c) Test 1 cohort; (d) Test 2 cohort. ROC, receiver operating characteristic; AUC, area under the curve; LR, logistic regression; SVM, support vector machine.

6. Handcrafted features

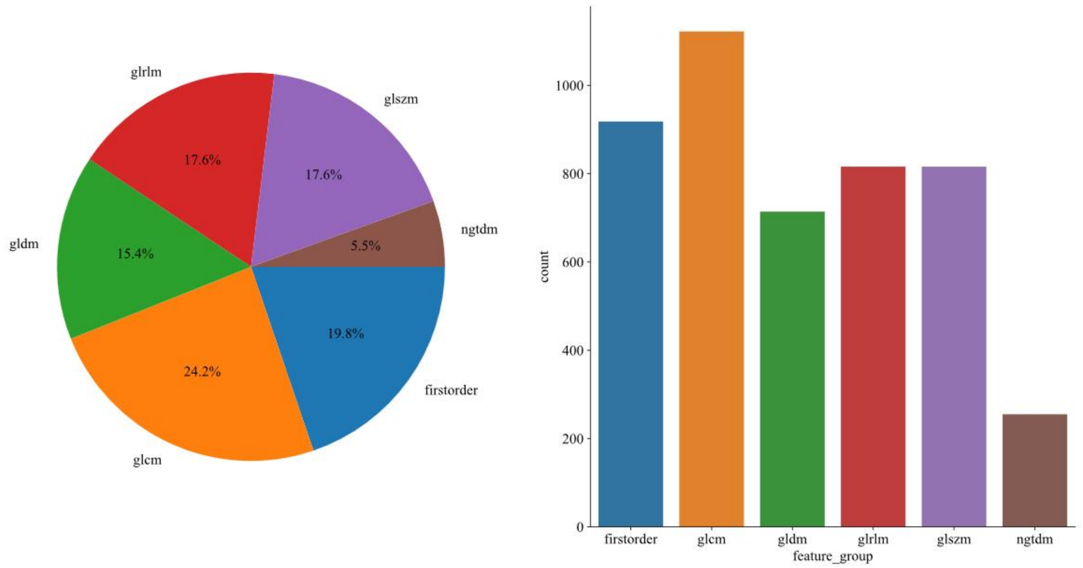

Supplementary Figure 3. Number and ratio of handcrafted features.

7. Details of radiomics models

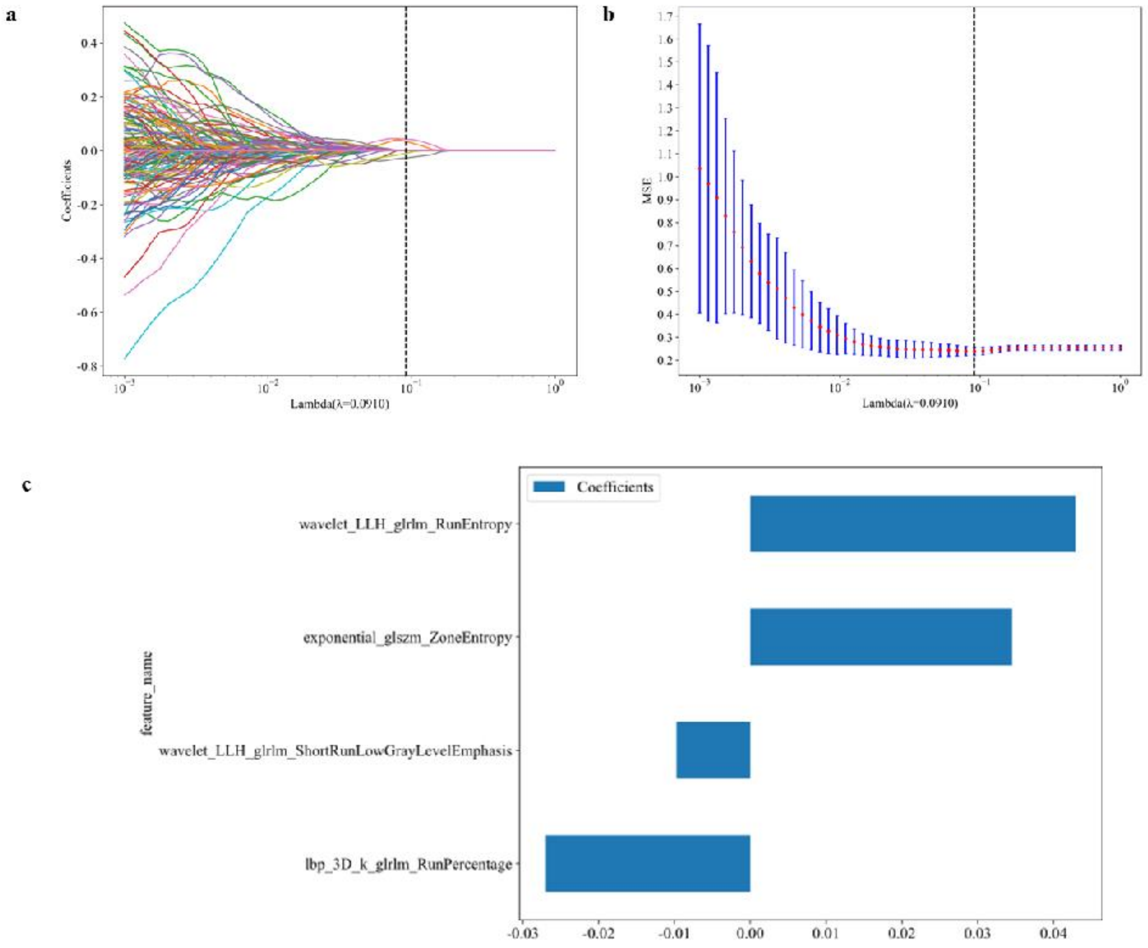

Supplementary Figure 4. LASSO-based feature selection within the radiomics model, optimized via 10-fold cross-validation (λ = 0.0910). (A) Coefficient trajectories across cross-validation folds. (B) MSE profile during cross-validation. (C) Distribution of Rad-scores derived from LASSO-retained features. LASSO, least absolute shrinkage and selection operator; MSE, mean standard error.

Supplementary Table 2. Performance of machine learning algorithms for radiomics model in each cohorts

Machine learning algorithms	Accuracy	AUC	95% CI	Sensitivity	Specificity	PPV	NPV	Cohort
LR	0.678	0.736	0.657 - 0.816	0.791	0.582	0.616	0.767	Train
LR	0.698	0.743	0.621 - 0.865	0.839	0.562	0.65	0.783	Internal validation
LR	0.772	0.716	0.544 - 0.888	0.588	0.85	0.625	0.829	Test 1
LR	0.696	0.732	0.641 - 0.823	0.611	0.85	0.88	0.548	Test 2
SVM	0.692	0.728	0.646 - 0.810	0.776	0.62	0.634	0.766	Train
SVM	0.667	0.719	0.591 - 0.846	0.903	0.437	0.609	0.824	Internal validation
SVM	0.702	0.682	0.506 - 0.859	0.588	0.75	0.5	0.811	Test 1
SVM	0.688	0.691	0.588 - 0.794	0.653	0.75	0.825	0.545	Test 2
RandomForest	0.747	0.776	0.700 - 0.852	0.851	0.658	0.679	0.839	Train
RandomForest	0.683	0.753	0.630 - 0.875	0.903	0.469	0.622	0.833	Internal validation
RandomForest	0.684	0.669	0.505 - 0.833	0.118	0.925	0.4	0.712	Test 1
RandomForest	0.598	0.68	0.580 - 0.780	0.486	0.8	0.814	0.464	Test 2
ExtraTrees	0.712	0.774	0.700 - 0.848	0.776	0.658	0.658	0.776	Train
ExtraTrees	0.73	0.744	0.623 - 0.866	0.903	0.562	0.667	0.857	Internal validation
ExtraTrees	0.737	0.72	0.554 - 0.886	0.353	0.9	0.6	0.766	Test 1
ExtraTrees	0.688	0.703	0.605 - 0.801	0.639	0.775	0.836	0.544	Test 2
XGBoost	0.699	0.804	0.736 - 0.872	0.403	0.949	0.871	0.652	Train
XGBoost	0.698	0.73	0.605 - 0.855	0.677	0.719	0.7	0.697	Internal validation
XGBoost	0.737	0.704	0.544 - 0.863	0.529	0.825	0.562	0.805	Test 1
XGBoost	0.625	0.688	0.589 - 0.787	0.542	0.775	0.812	0.484	Test 2
LightGBM	0.829	0.892	0.841 - 0.942	0.761	0.886	0.85	0.814	Train
LightGBM	0.714	0.739	0.614 - 0.864	0.71	0.719	0.71	0.719	Internal validation
LightGBM	0.754	0.708	0.541 - 0.875	0.588	0.825	0.588	0.825	Test 1
LightGBM	0.652	0.654	0.552 - 0.756	0.667	0.625	0.762	0.51	Test 2

Abbreviation: AUC: area under the curve; CI: confidence interval; PPV: positive predictive value; NPV: negative predictive value; LR, logistic regression; SVM, support vector machine.

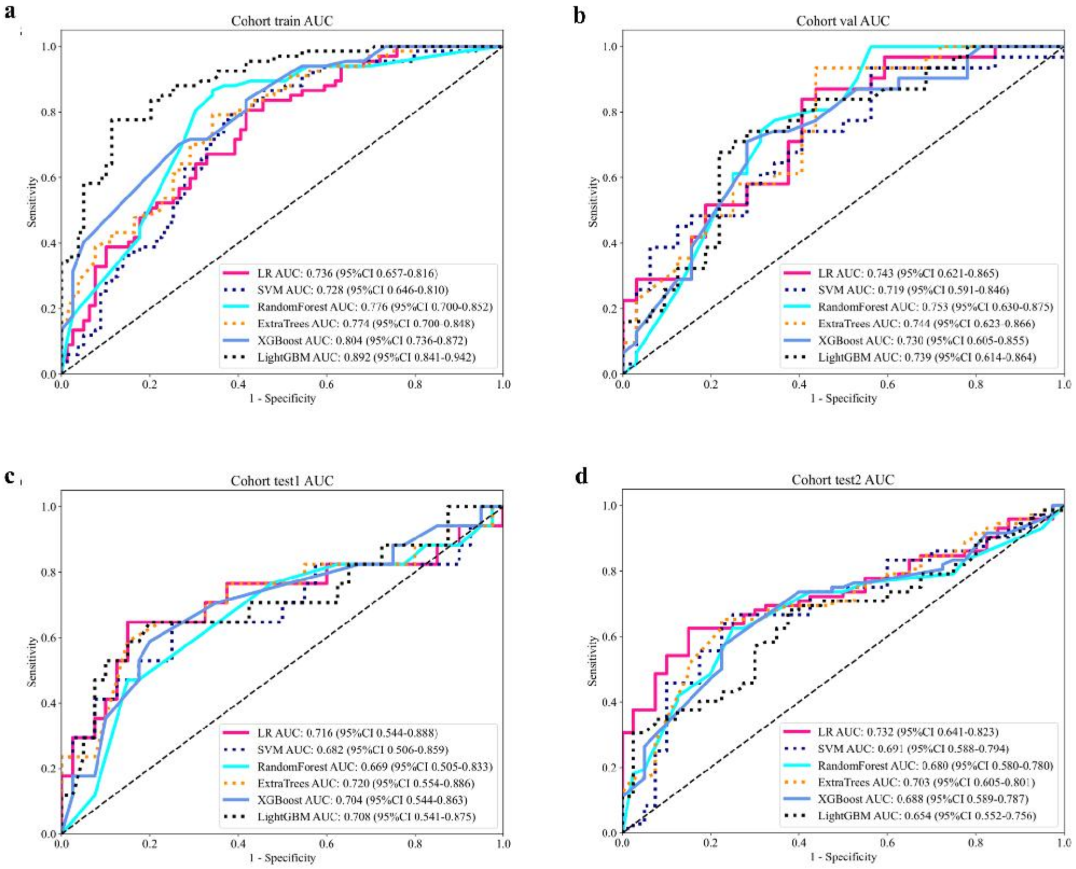

Supplementary Figure 5. ROC curves of multiple machine learning algorithms of different radiomics models across four independent cohorts. The figure illustrates the discriminatory performance of six distinct classifiers, with AUC values and corresponding 95% CIs provided for each model. The algorithms evaluated include: LR, SVM, RandomForest, Extra Trees, XGBoost, and LightGBM. (a) Training cohort; (b) Internal validation cohort; (c) Test 1 cohort; (d) Test 2 cohort. ROC, receiver operating characteristic; AUC, area under the curve; LR, logistic regression; SVM, support vector machine.

8. Details of peritumoral models

Peri-1mm

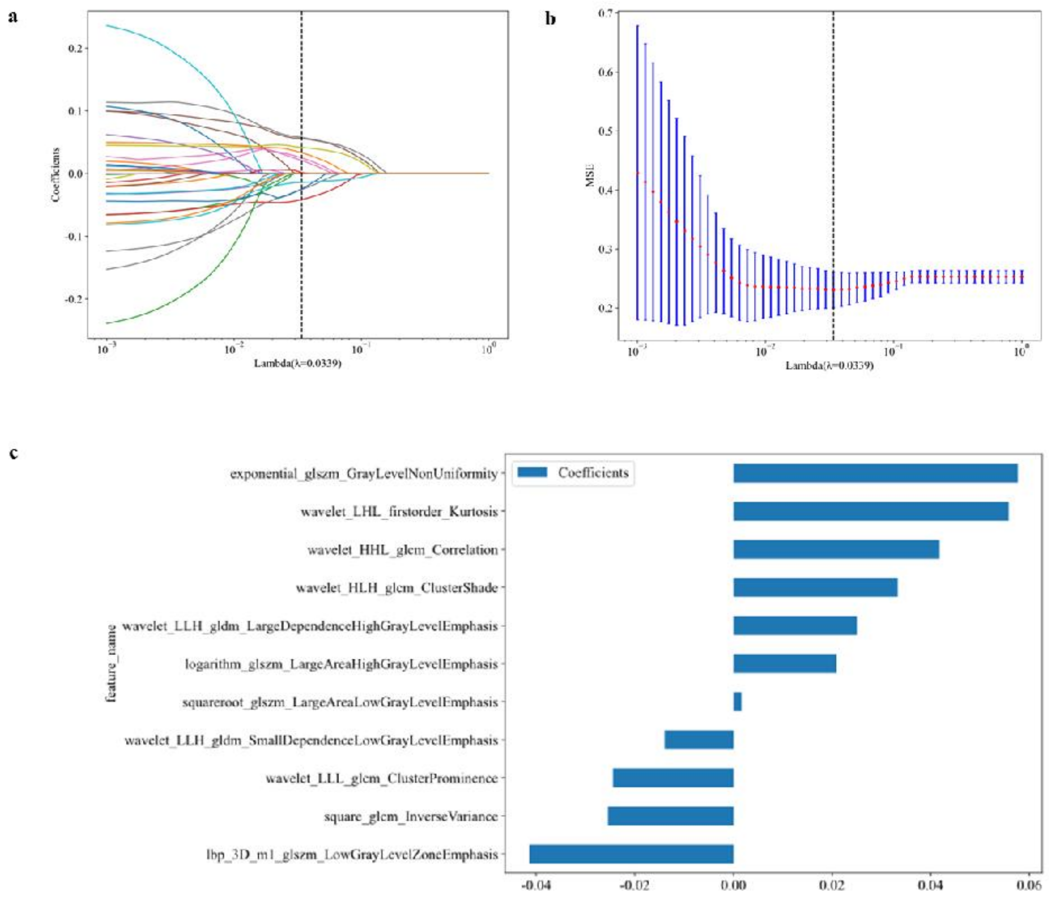

Supplementary Figure 6. LASSO-based feature selection within the Pei-1mm model, optimized via 10-fold cross-validation (λ = 0.0339). (A) Coefficient trajectories across cross-validation folds. (B) MSE profile during cross-validation. (C) Distribution of Rad-scores derived from LASSO-retained features. LASSO, least absolute shrinkage and selection operator; MSE, Mean Standard Error.

Supplementary Table 3. Performance of machine learning algorithms for Peri-1mm model in each cohorts

Machine learning algorithms	Accuracy	AUC	95% CI	Sensitivity	Specificity	PPV	NPV	Cohort
LR	0.733	0.779	0.704 - 0.854	0.493	0.937	0.868	0.685	Train
LR	0.651	0.698	0.566 - 0.829	0.871	0.437	0.6	0.778	Internal validation
LR	0.667	0.678	0.517 - 0.839	0.588	0.7	0.455	0.8	Test 1
LR	0.652	0.589	0.477 - 0.701	0.792	0.4	0.704	0.516	Test 2
SVM	0.74	0.783	0.707 - 0.859	0.597	0.861	0.784	0.716	Train
SVM	0.698	0.743	0.621 - 0.865	0.806	0.594	0.658	0.76	Internal validation
SVM	0.632	0.609	0.447 - 0.771	0.647	0.625	0.423	0.806	Test 1
SVM	0.652	0.687	0.582 - 0.793	0.611	0.725	0.8	0.509	Test 2
RandomForest	0.637	0.763	0.692 - 0.833	0.239	0.975	0.889	0.602	Train
RandomForest	0.619	0.836	0.744 - 0.927	0.226	1	1	0.571	Internal validation
RandomForest	0.702	0.646	0.481 - 0.810	0	1	0	0.702	Test 1
RandomForest	0.473	0.666	0.567 - 0.764	0.25	0.875	0.783	0.393	Test 2
ExtraTrees	0.774	0.838	0.774 - 0.901	0.716	0.823	0.774	0.774	Train
ExtraTrees	0.714	0.77	0.654 - 0.885	0.935	0.5	0.644	0.889	Internal validation
ExtraTrees	0.737	0.67	0.512 - 0.828	0.353	0.9	0.6	0.766	Test 1
ExtraTrees	0.67	0.654	0.546 - 0.763	0.681	0.65	0.778	0.531	Test 2
XGBoost	0.774	0.857	0.798 - 0.916	0.627	0.899	0.84	0.74	Train
XGBoost	0.746	0.794	0.684 - 0.903	0.839	0.656	0.703	0.808	Internal validation
XGBoost	0.561	0.652	0.499 - 0.806	0.824	0.45	0.389	0.857	Test 1
XGBoost	0.643	0.614	0.501 - 0.727	0.764	0.425	0.705	0.5	Test 2
LightGBM	0.76	0.852	0.792 - 0.913	0.746	0.772	0.735	0.782	Train
LightGBM	0.746	0.803	0.695 - 0.911	0.742	0.75	0.742	0.75	Internal validation
LightGBM	0.526	0.619	0.464 - 0.775	0.824	0.4	0.368	0.842	Test 1
LightGBM	0.661	0.679	0.573 - 0.785	0.639	0.7	0.793	0.519	Test 2

Abbreviation: AUC: area under the curve; CI: confidence interval; PPV: positive predictive value; NPV: negative predictive value; LR, logistic regression; SVM, support vector machine.

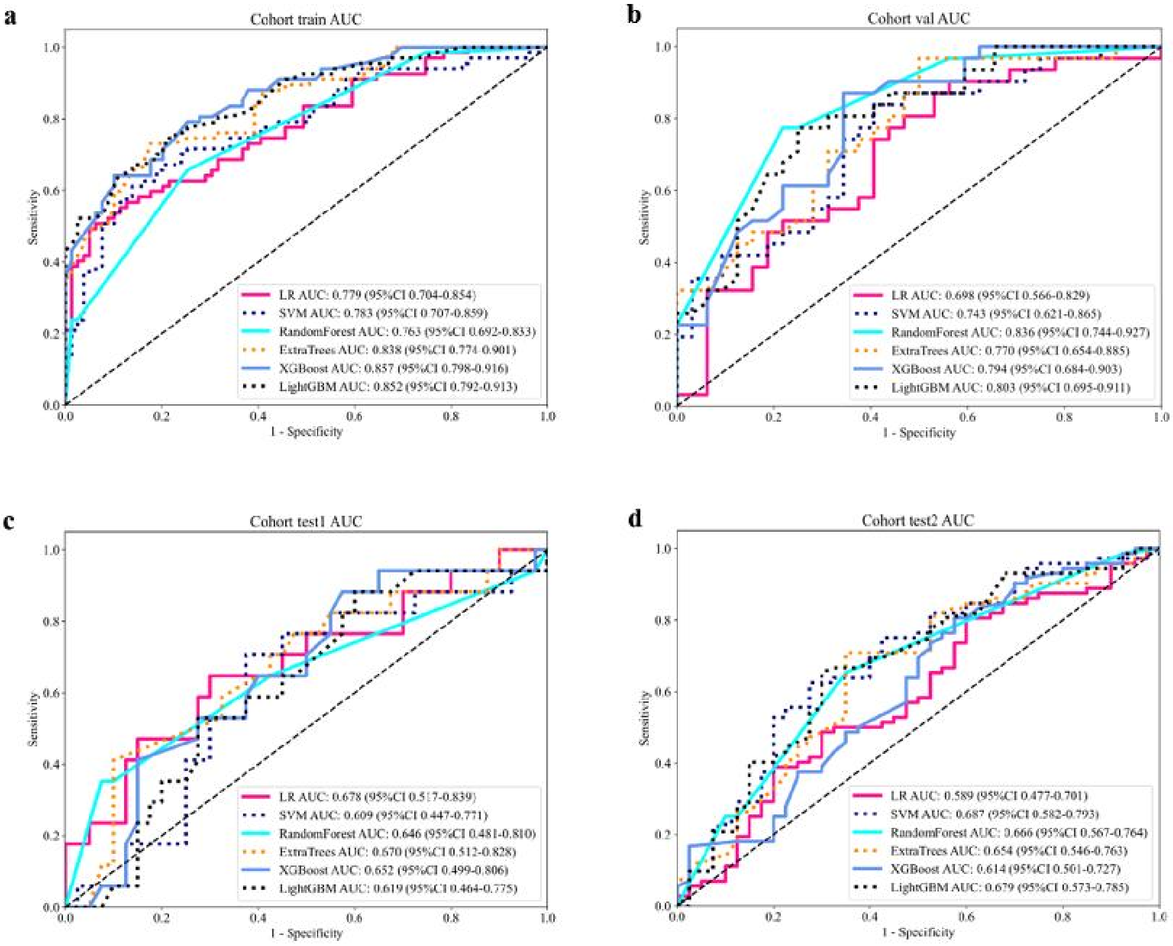

Supplementary Figure 7. ROC curves of multiple machine learning algorithms of Peri-1mm models across four independent cohorts. The figure illustrates the discriminatory performance of six distinct classifiers, with AUC values and corresponding 95% CIs provided for each model. The algorithms evaluated include: LR, SVM, RandomForest, Extra Trees, XGBoost, and LightGBM. (a) Training cohort; (b) Internal validation cohort; (c) Test 1 cohort; (d) Test 2 cohort. ROC, receiver operating characteristic; AUC, area under the curve; LR, logistic regression; SVM, support vector machine.

Peri-2mm

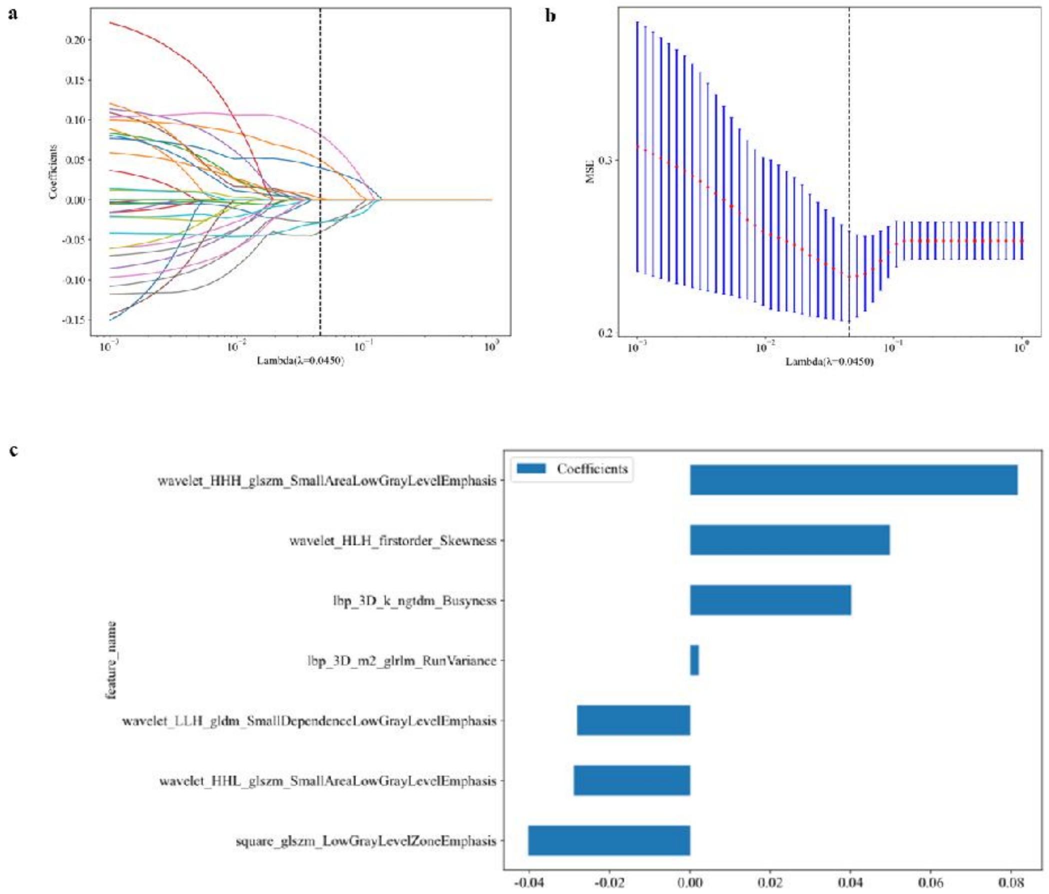

Supplementary Figure 8. LASSO-based feature selection within the Pei-2mm model, optimized via 10-fold cross-validation (λ = 0.0450). (A) Coefficient trajectories across cross-validation folds. (B) MSE profile during cross-validation. (C) Distribution of Rad-scores derived from LASSO-retained features. LASSO, least absolute shrinkage and selection operator; MSE, Mean Standard Error.

Supplementary Table 4. Performance of machine learning algorithms for Peri-2mm model in each cohorts

Machine learning algorithms	Accuracy	AUC	95% CI	Sensitivity	Specificity	PPV	NPV	Cohort
LR	0.726	0.758	0.680 - 0.836	0.552	0.873	0.787	0.697	Train
LR	0.651	0.653	0.516 - 0.791	0.484	0.812	0.714	0.619	Internal validation
LR	0.702	0.606	0.428 - 0.784	0.412	0.825	0.5	0.767	Test 1
LR	0.714	0.671	0.565 - 0.777	0.875	0.425	0.733	0.654	Test 2
SVM	0.781	0.837	0.772 - 0.903	0.716	0.835	0.787	0.776	Train
SVM	0.683	0.704	0.570 - 0.837	0.806	0.562	0.641	0.75	Internal validation
SVM	0.719	0.57	0.378 - 0.762	0.353	0.875	0.545	0.761	Test 1
SVM	0.688	0.659	0.551 - 0.766	0.833	0.425	0.723	0.586	Test 2
RandomForest	0.774	0.837	0.773 - 0.901	0.791	0.759	0.736	0.811	Train
RandomForest	0.746	0.794	0.684 - 0.903	0.742	0.75	0.742	0.75	Internal validation
RandomForest	0.684	0.656	0.482 - 0.830	0.588	0.725	0.476	0.806	Test 1
RandomForest	0.679	0.701	0.600 - 0.802	0.722	0.6	0.765	0.545	Test 2
ExtraTrees	0.747	0.814	0.746 - 0.882	0.731	0.759	0.721	0.769	Train
ExtraTrees	0.683	0.748	0.627 - 0.869	0.871	0.5	0.628	0.8	Internal validation
ExtraTrees	0.684	0.607	0.429 - 0.785	0.588	0.725	0.476	0.806	Test 1
ExtraTrees	0.661	0.669	0.565 - 0.772	0.681	0.625	0.766	0.521	Test 2
XGBoost	0.801	0.878	0.825 - 0.931	0.806	0.797	0.771	0.829	Train
XGBoost	0.651	0.726	0.602 - 0.850	0.355	0.937	0.846	0.6	Internal validation
XGBoost	0.772	0.63	0.446 - 0.815	0.353	0.95	0.75	0.776	Test 1
XGBoost	0.67	0.691	0.588 - 0.794	0.708	0.6	0.761	0.533	Test 2
LightGBM	0.541	0.769	0.695 - 0.843	0	1	0	0.541	Train
LightGBM	0.635	0.713	0.593 - 0.833	0.613	0.656	0.633	0.636	Internal validation
LightGBM	0.544	0.567	0.417 - 0.717	0.588	0.525	0.345	0.75	Test 1
LightGBM	0.357	0.679	0.581 - 0.777	0	1	0	0.357	Test 2

Abbreviation: AUC: area under the curve; CI: confidence interval; PPV: positive predictive value; NPV: negative predictive value; LR, logistic regression; SVM, support vector machine.

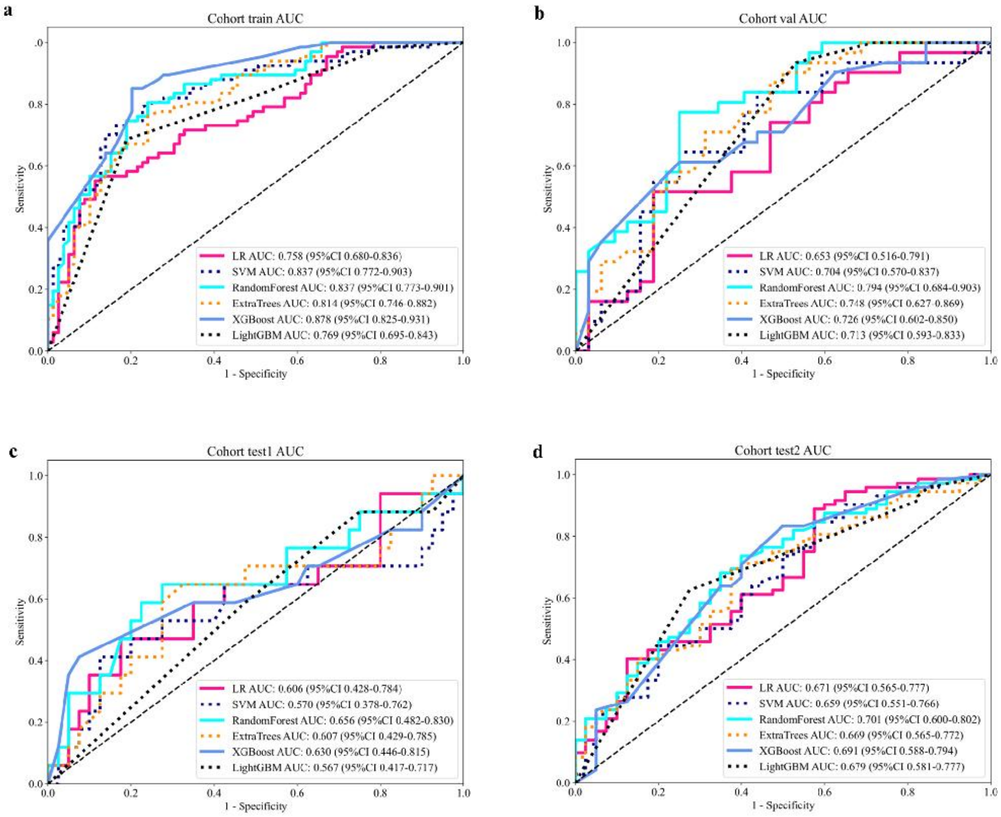

Supplementary Figure 9. ROC curves of multiple machine learning algorithms of Peri-2mm models across four independent cohorts. The figure illustrates the discriminatory performance of six distinct classifiers, with AUC values and corresponding 95% CIs provided for each model. The algorithms evaluated include: LR, SVM, RandomForest, Extra Trees, XGBoost, and LightGBM. (a) Training cohort; (b) Internal validation cohort; (c) Test 1 cohort; (d) Test 2 cohort. ROC, receiver operating characteristic; AUC, area under the curve; LR, logistic regression; SVM, support vector machine.

Peri-3mm

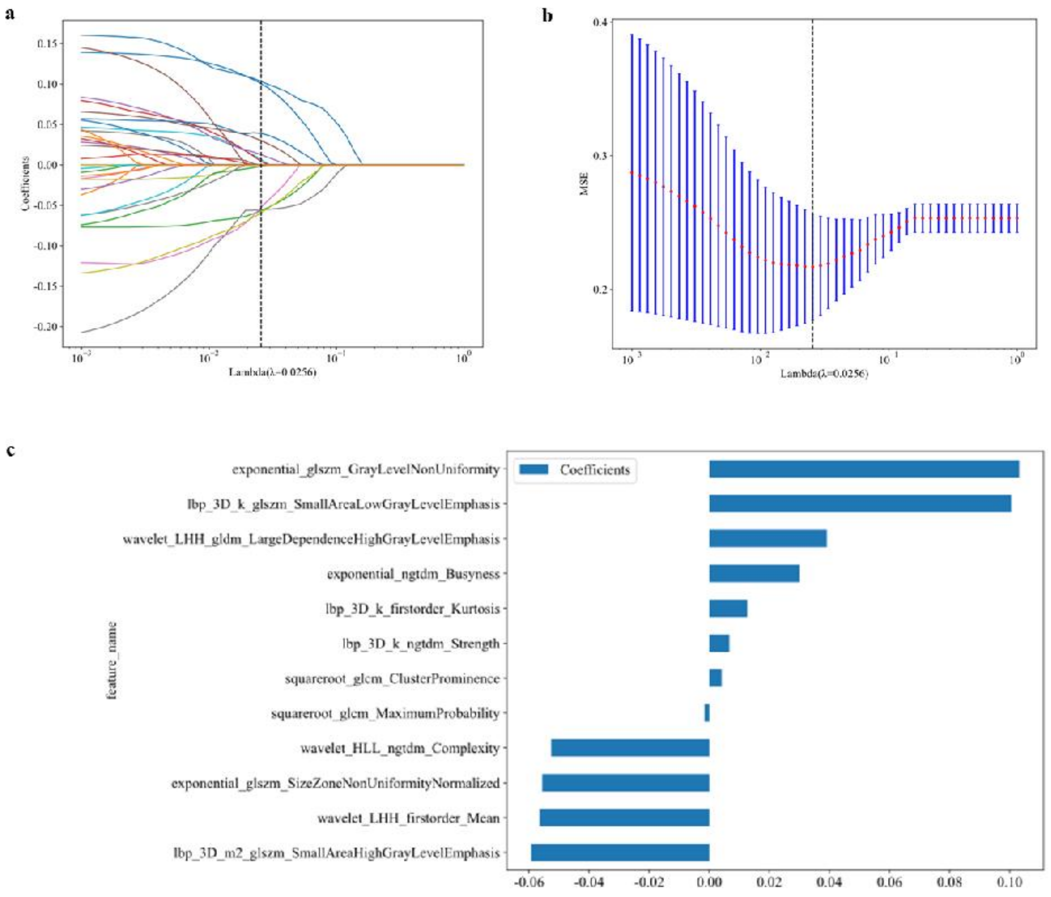

Supplementary Figure 10. LASSO-based feature selection within the Pei-3mm model, optimized via 10-fold cross-validation (λ = 0.0256). (A) Coefficient trajectories across cross-validation folds. (B) MSE profile during cross-validation. (C) Distribution of Rad-scores derived from LASSO-retained features. LASSO, least absolute shrinkage and selection operator; MSE, mean standard error.

Supplementary Table 5. Performance of machine learning algorithms for Peri-3mm model in each cohorts

Machine learning algorithms	Accuracy	AUC	95% CI	Sensitivity	Specificity	PPV	NPV	Cohort
LR	0.774	0.812	0.741 - 0.882	0.627	0.899	0.84	0.74	Train
LR	0.778	0.787	0.669 - 0.906	0.806	0.75	0.758	0.8	Internal validation
LR	0.561	0.644	0.475 - 0.813	0.765	0.475	0.382	0.826	Test 1
LR	0.661	0.606	0.495 - 0.716	0.806	0.4	0.707	0.533	Test 2
SVM	0.726	0.812	0.744 - 0.880	0.896	0.582	0.645	0.868	Train
SVM	0.762	0.806	0.694 - 0.918	0.903	0.625	0.7	0.87	Internal validation
SVM	0.649	0.649	0.483 - 0.815	0.765	0.6	0.448	0.857	Test 1
SVM	0.562	0.542	0.430 - 0.654	0.569	0.55	0.695	0.415	Test 2
RandomForest	0.747	0.836	0.772 - 0.899	0.791	0.709	0.697	0.8	Train
RandomForest	0.746	0.812	0.709 - 0.916	0.935	0.562	0.674	0.9	Internal validation
RandomForest	0.596	0.674	0.523 - 0.824	0.706	0.55	0.4	0.815	Test 1
RandomForest	0.625	0.722	0.627 - 0.816	0.528	0.8	0.826	0.485	Test 2
ExtraTrees	0.788	0.87	0.813 - 0.927	0.836	0.747	0.737	0.843	Train
ExtraTrees	0.73	0.779	0.663 - 0.895	0.774	0.687	0.706	0.759	Internal validation
ExtraTrees	0.632	0.672	0.513 - 0.831	0.765	0.575	0.433	0.852	Test 1
ExtraTrees	0.545	0.637	0.529 - 0.745	0.375	0.85	0.818	0.43	Test 2
XGBoost	0.747	0.843	0.780 - 0.906	0.701	0.785	0.734	0.756	Train
XGBoost	0.746	0.815	0.710 - 0.919	0.871	0.625	0.692	0.833	Internal validation
XGBoost	0.596	0.712	0.562 - 0.861	0.588	0.6	0.385	0.774	Test 1
XGBoost	0.536	0.605	0.494 - 0.716	0.389	0.8	0.778	0.421	Test 2
LightGBM	0.753	0.815	0.747 - 0.883	0.672	0.823	0.763	0.747	Train
LightGBM	0.73	0.808	0.703 - 0.914	0.871	0.594	0.675	0.826	Internal validation
LightGBM	0.702	0.668	0.511 - 0.824	0.412	0.825	0.5	0.767	Test 1
LightGBM	0.67	0.627	0.517 - 0.736	0.833	0.375	0.706	0.556	Test 2

Abbreviation: AUC: area under the curve; CI: confidence interval; PPV: positive predictive value; NPV: negative predictive value; LR, logistic regression; SVM, support vector machine.

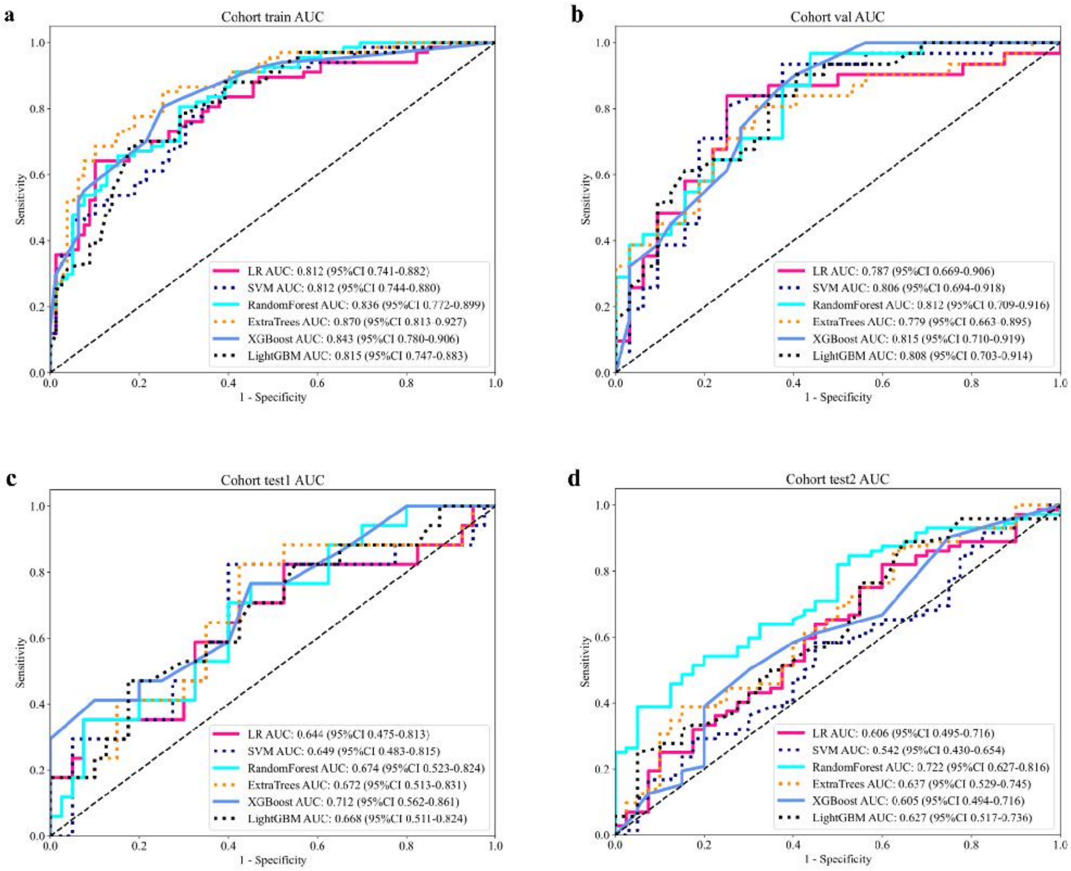

Supplementary Figure 11. ROC curves of multiple machine learning algorithms of Peri-3mm models across four independent cohorts. The figure illustrates the discriminatory performance of six distinct classifiers, with AUC values and corresponding 95% CIs provided for each model. The algorithms evaluated include: LR, SVM, RandomForest, Extra Trees, XGBoost, and LightGBM. (a) Training cohort; (b) Internal validation cohort; (c) Test 1 cohort; (d) Test 2 cohort. ROC, receiver operating characteristic; AUC, area under the curve; LR, logistic regression; SVM, support vector machine.

9. Details of habitat imaging models

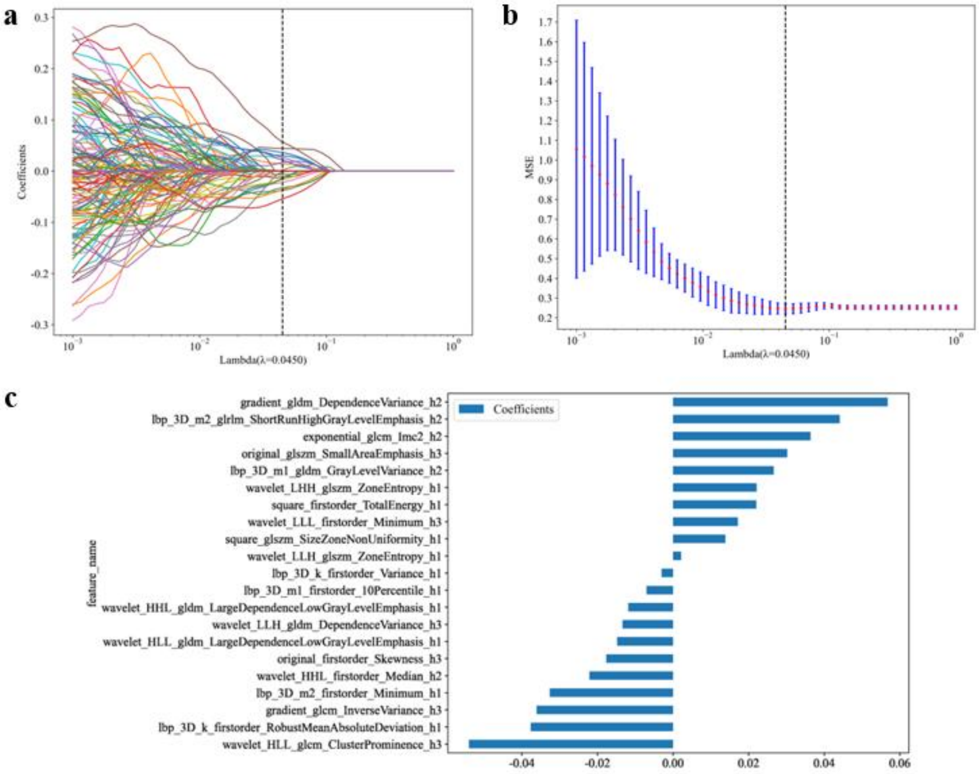

Supplementary Figure 12. LASSO-based feature selection within the habitat imaging models, optimized via 10-fold cross-validation (λ = 0.0256). (A) Coefficient trajectories across cross-validation folds. (B) MSE profile during cross-validation. (C) Distribution of Rad-scores derived from LASSO-retained features. LASSO, least absolute shrinkage and selection operator; MSE, mean standard error.

Supplementary Table 6. Performance of machine learning algorithms for habitat model in each cohorts

Machine learning algorithms	Accuracy	AUC	95% CI	Sensitivity	Specificity	PPV	NPV	Cohort
LR	0.822	0.875	0.820 - 0.931	0.881	0.772	0.766	0.884	Train
LR	0.683	0.733	0.607 - 0.859	0.742	0.625	0.657	0.714	Internal validation
LR	0.719	0.747	0.611 - 0.883	0.706	0.725	0.522	0.853	Test 1
LR	0.491	0.534	0.427 - 0.641	0.25	0.925	0.857	0.407	Test 2
SVM	0.795	0.867	0.810 - 0.924	0.627	0.937	0.894	0.747	Train
SVM	0.683	0.739	0.618 - 0.861	0.677	0.687	0.677	0.687	Internal validation
SVM	0.544	0.609	0.443 - 0.775	0.706	0.475	0.364	0.792	Test 1
SVM	0.661	0.569	0.452 - 0.686	0.764	0.475	0.724	0.528	Test 2
RandomForest	0.788	0.844	0.782 - 0.906	0.821	0.759	0.743	0.833	Train
RandomForest	0.714	0.754	0.633 - 0.875	0.581	0.844	0.783	0.675	Internal validation
RandomForest	0.667	0.75	0.615 - 0.885	0.647	0.675	0.458	0.818	Test 1
RandomForest	0.705	0.772	0.685 - 0.859	0.611	0.875	0.898	0.556	Test 2
ExtraTrees	0.747	0.817	0.749 - 0.884	0.821	0.684	0.687	0.818	Train
ExtraTrees	0.746	0.775	0.658 - 0.893	0.581	0.906	0.857	0.69	Internal validation
ExtraTrees	0.754	0.705	0.556 - 0.854	0.353	0.925	0.667	0.771	Test 1
ExtraTrees	0.482	0.602	0.494 - 0.711	0.222	0.95	0.889	0.404	Test 2
XGBoost	0.815	0.9	0.852 - 0.947	0.91	0.734	0.744	0.906	Train
XGBoost	0.794	0.886	0.808 - 0.964	0.613	0.969	0.95	0.721	Internal validation
XGBoost	0.719	0.82	0.712 - 0.928	0.882	0.65	0.517	0.929	Test 1
XGBoost	0.634	0.804	0.725 - 0.884	0.431	1	1	0.494	Test 2
LightGBM	0.76	0.866	0.810 - 0.923	0.642	0.861	0.796	0.739	Train
LightGBM	0.746	0.784	0.665 - 0.903	0.806	0.687	0.714	0.786	Internal validation
LightGBM	0.737	0.779	0.660 - 0.898	0.647	0.775	0.55	0.838	Test 1
LightGBM	0.696	0.739	0.634 - 0.844	0.722	0.65	0.788	0.565	Test 2

Abbreviation: AUC: area under the curve; CI: confidence interval; PPV: positive predictive value; NPV: negative predictive value; LR, logistic regression; SVM, support vector machine.

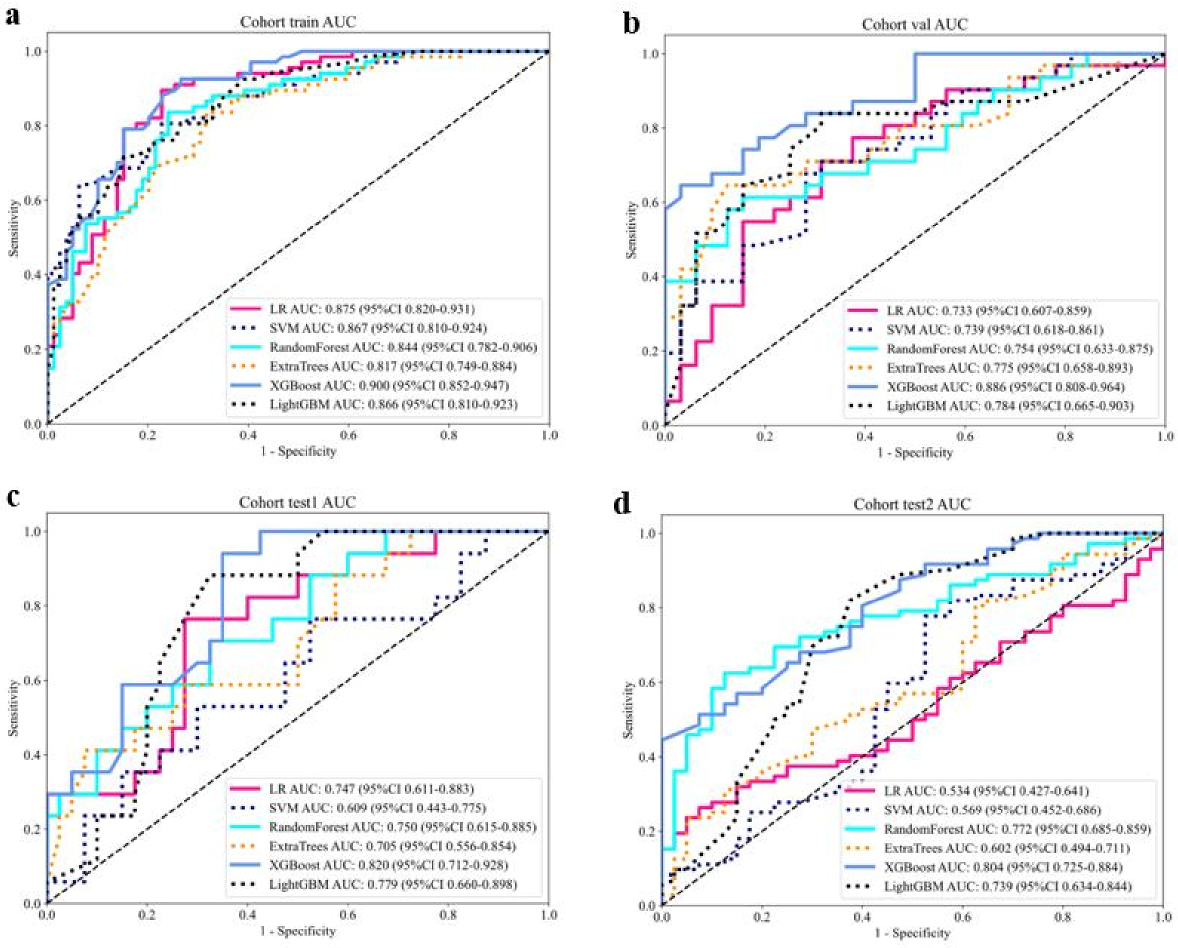

Supplementary Figure 13. ROC curves of multiple machine learning algorithms of habitat imaging models across four independent cohorts. The figure illustrates the discriminatory performance of six distinct classifiers, with AUC values and corresponding 95% CIs provided for each model. The algorithms evaluated include: LR, SVM, RandomForest, Extra Trees, XGBoost, and LightGBM. (a) Training cohort; (b) Internal validation cohort; (c) Test 1 cohort; (d) Test 2 cohort. ROC, receiver operating characteristic; AUC, area under the curve; LR, logistic regression; SVM, support vector machine.
